# Supplementary material for: Liquid-liquid reactions performed by cellular reactors
Source: Nat Commun. 2024 Jul 3;15:5579. doi: 10.1038/s41467-024-49953-z (PMC11222485; doi:10.1038/s41467-024-49953-z)
Supplement: Supplementary file 1 — Supporting Information [file 41467_2024_49953_MOESM1_ESM.pdf]

Supporting Information for Liquid-Liquid Reactions Performed by Cellular Reactors

Jinzhe Cao[1], Shengyang Tao[1,2,3,4]*

1: School of Chemistry, Dalian University of Technology, 116024, Dalian, Liaoning, China

2: State Key Laboratory of Fine Chemicals, Dalian University of Technology, 116024, Dalian, Liaoning, China

3: Frontier Science Center for Smart Materials, Dalian University of Technology, 116024, Dalian, Liaoning, China

4：Dalian Key Laboratory of Intelligent Chemistry, Dalian University of Technology, Dalian 116024, Liaoning, China

E-mail: taosy@dlut.edu.cn

Catalog

Materials and characterisation 4
Reagents 4
Substrates and products analysis 4
Theoretical analysis and functional demonstration of cellular reactor 7
Supplementary Figure 1. 7
Supplementary Figure 2. 9
Supplementary Figure 3. 10
Supplementary Table 1. 11
Supplementary Table 2. 13
Supplementary Table 3. 13
Supplementary Figure 4. 14
Supplementary Table 4. 16
Supplementary Figure 5. 17
Supplementary Table 5. 18
Supplementary Table 6. 19
Molecular dynamics simulation 20
Modeling of interfacial reactions. 21
Supplementary Figure 6. 22
Supplementary Figure 7. 24
Supplementary Figure 8. 26
GC-MS, UPLC-MS and NMR Data 27
Supplementary Figure 9. 27
Supplementary Figure 10. 28
Supplementary Figure 11. 29
Supplementary Figure 12. 30
Supplementary Table 7. 31
Supplementary Figure 13. 32
Supplementary Figure 14. 33
Supplementary Figure 15. 34
Supplementary Figure 16. 35
Supplementary Figure 17. 36
Supplementary Figure 18. 37
Supplementary Figure 19. 38
Supplementary Figure 20. 39
Supplementary Figure 21. 40
Supplementary Figure 22. 41
Supplementary Figure 23. 42
Supplementary Figure 24. 43
Supplementary Figure 25. 44
Supplementary Figure 26. 45

Supplementary Figure 27. 46
Supplementary Figure 28. 47
Supplementary Figure 29. 48
Supplementary Figure 30. 49
Supplementary Figure 31. 50
Supplementary Figure 32. 51
Supplementary Figure 33. 52
Cyclicity and stability of cellular reactor 53
Supplementary Table 8. 53
Supplementary Figure 34. 54
Supplementary Table 9. 55
Supplementary Table 10 56
Supplementary Figure 35. 58
Supplementary Figure 36. 59
Supplementary Figure 37. 60
Supplementary Figure 38. 61
Supplementary Figure 39. 62
Supplementary Table 11. 64
Particle Image Velocimetry 65
Supplementary Figure 40. 65
Supplementary Table 12 65
Supplementary Figure 41. 66
Supplementary Table 13 67
Effect of rotation speed on reaction rate 69
Supplementary Table 14 69
Simulation experiments with solid additives 71
Supplementary Table 15 71
Cross-contamination between water phase and organic phase 72
Supplementary Table 16 73
Supplementary Table 17 74
Supplementary Figure 42. 76
Supplementary Table 18 77
Comparison between the use and non-use of cellular reactor 79
Supplementary Table 19 81
SI Reference 83

Materials and characterisation

Reagents

Bromothymol blue (AR), 3,4-2H-dihydropyran (AR), benzyl alcohol (AR), 1-octanol (AR), 1-hexanol (AR), cyclohexanol (AR), potassium carbonate (AR), benzaldehyde (AR), 1H,1H,2H,2H-perfluorodecyltrimethoxysilane malononitrile (AR), 3-chlorobenzaldehyde (AR), 4-chlorobenzaldehyde (AR), 4-bromobenzaldehyde (AR), 2-phenylethanethiol (AR), 4-toluenethiol (AR), diiodomethane (AR) and titrant-component for volumetric Karl Fischer titration(A: solvent, B: titrant) were purchased from the Shanghai Aladdin Biochemical Technology Co., Ltd. Sulfuric acid (H_2SO_4, AR), sodium hydroxide (NaOH, AR)，sudan III, methylene Blue trihydrate and hydrogen peroxide were purchased from the Sinopharm Chemical Regent Co. Ltd. Thioanisole (AR), tetrahydrothiophene (AR), 1-hexanethiol (AR), 4-fluorothiophenol (AR), dibutyl sulfide (AR), (allylthio)benzene (AR), 4-Nitrothioanisole (AR) and acetonitrile (HPLC-MS) were purchased from the Adamas Reagents Ltd. Isopropyl alcohol (AR) was purchased from the Tianjin Komiou Chemical Reagent Co. Ltd. All the reagents were used directly without further purification. Deionized water was used in all experiments.

Substrates and products analysis

All reaction substrates, and Agilent GC-MS measured product concentrations, conversions, and yields were calculated from these. Gas chromatography-mass

spectrometry (GC-MS, Agilent 8890/5977B) with an HP-5MS UI capillary column was used to determine the composition of the product, with helium used as the carrier gas at a flow rate of 1 mL/min. Injection volume is 0.1 μL. The following mass spectrometry conditions were used: transmission-line temperature, 280 °C; ion-source temperature, 230 °C; quadrupole temperature, 150 °C.

Reactions involving or producing non-volatile compounds were tested using a Waters ACQUITY UPLC H-Class PLUS Core System ultra-high performance liquid chromatography-mass spectrometry (UPLC-MS) equipped with a PDA detector and an ion source. Mass spectra of the compounds were obtained, and conversion rates were calculated based on the chromatograms.

Addition reaction of alcohols to 3,4-2H-dihydropyran

The bypass ratio was 100:1. The inlet temperature was set to 300 °C. The initial column temperature was set to 70 °C, programmed to heat to 84 °C at 3 °C/min, programmed to heat to 240 °C at 30 °C/min, and maintained there for 2 min.

Knoevenagel condensation reaction

The bypass ratio was 100:1. The inlet temperature was set to 330 °C. The initial column temperature was set to 100 °C, programmed to heat to 310 °C at 30 °C/min, and maintained there for 1 min.

Coupling reactions of sulfhydryl compounds

The bypass ratio was 30:1. The inlet temperature was set to 330 °C. The initial column temperature was set to 70 °C, programmed to heat to 315 °C at 30 °C/min, and maintained there for 1 min.

Oxidation reaction of thioethers

The bypass ratio was 30:1. The inlet temperature was set to 355°C. The initial column temperature was set to 70 °C, programmed to heat to 315 °C at 30 °C/min, and maintained there for 1 min.

Reactions involving or generating compounds that are difficult to volatilize were tested using the Waters ACQUITY UPLC H-Class PLUS Core System Ultra High-Pressure Liquid Chromatography Mass Spectrometry (UPLC-MS) equipped with a PDA detector and a SQ Detector 2 single quadrupole mass spectrometer. The test was performed in positive ion mode at a flow rate of 0.2 mL/min with a mobile phase composition of acetonitrile: water = 20:80, to which 0.1% formic acid was added. The injection volume for mass spectrometry was 0.1 μL, and the sample volume for chromatography was 5 μL.

Theoretical analysis and functional demonstration of cellular reactor

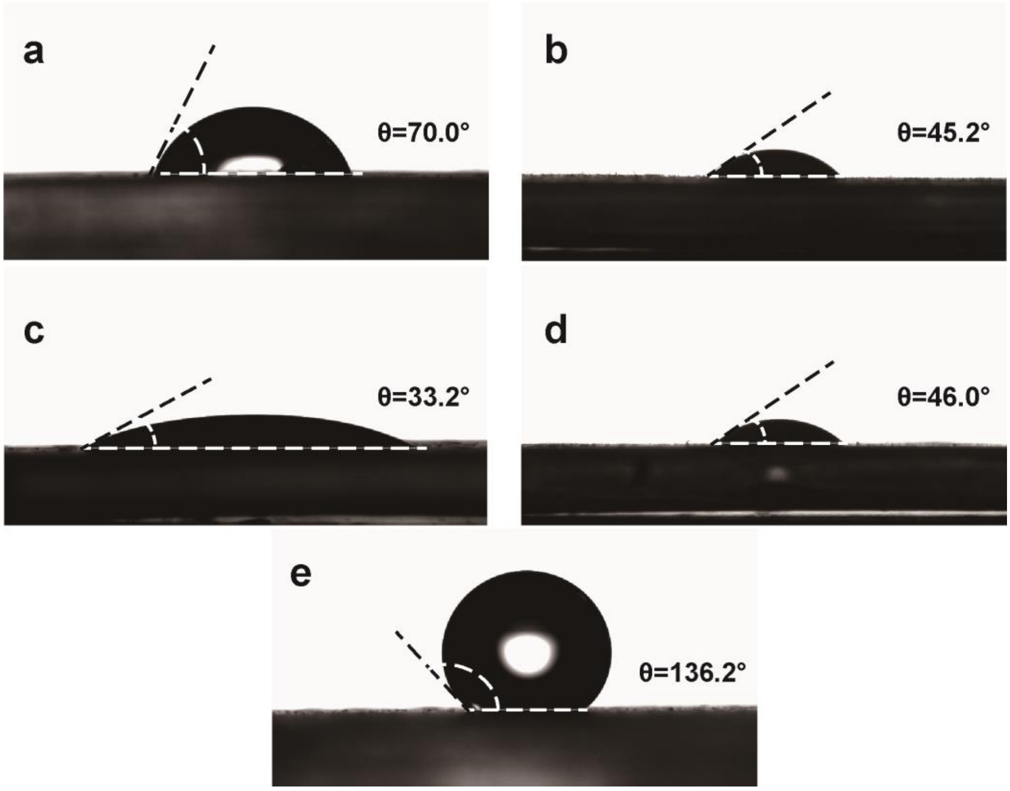

Supplementary Figure 1. Contact angle images of different surface treatments (a) Pristine surface (water contact angle) (b) Pristine surface (diiodomethane contact angle) (c) Oxygen plasma treated surface (water contact angle) (d) Oxygen plasma treated surface (diiodomethane contact angle) (a) Oxygen plasma-silane grafted surface (water contact angle)

In order to accurately characterize the surface wettability and its changes, flat plate structures were printed and post-treated using the same post-treatment as the reactor. After oxygen plasma treatment, the hydrophilic character of the surface was enhanced, and the contact angle was reduced from 70.0° to 33.2° Then, after gas-

phase chemical deposition of silane grafts, the surface took to a hydrophobic state and the contact angle changed to 136.2°.

The contact angle of diiodomethane on the surface was also measured in order to calculate the surface tension between the surface and air.

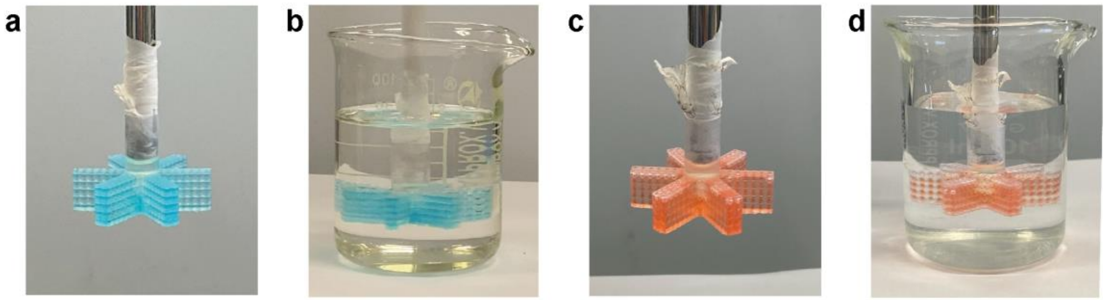

Supplementary Figure 2. Demonstration of the effect of holding the liquid in the cellular reactor (a) Holding water in the air (b) Holding water in cyclohexane (c) Holding cyclohexane in the air (d) Holding cyclohexane in water Water is colored with methylene blue and cyclohexane is colored with Sudan III. All reactor types are Reactor-C in Supplementary Table4.

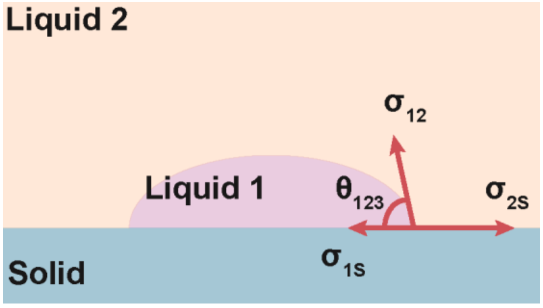

Supplementary Figure 3. Schematic diagram of the three-phase contact angle between liquid 1, liquid 2, and solid

The procedure for measuring and calculating the three-phase contact angle of the cellular reactor truss prosessed by O_2 plasma with water and cyclohexane was as follows, with the test environment at 20°C. First, the solid truss surface tension was measured. The solid surface tension can be regarded as the sum of dispersive surface tension and polar surface tension：

$$\sigma_s = \sigma_s^d + \sigma_s^p \quad (1)$$

where σ_s is the surface tension of the solid, σ_s^d is the dispersive surface tension of the solid and σ_s^p is the polar surface tension of the solid.

The interfacial tension of solid-liquid can be expressed as

$$\sigma_{sl} = \sigma_s + \sigma_l - 2\sqrt{\sigma_s^d \sigma_l^d} - 2\sqrt{\sigma_s^p \sigma_l^p} \quad (2)$$

where σ_{sl} represents the interfacial tension of the solid-liquid, σ_l represents the surface tension of the liquid, σ_l^d represents the dispersive surface tension of the liquid and σ_l^p represents the polar surface tension of the liquid.

The Young equation for the solid-liquid-gas three-phase is given by:

$$\sigma_s = \sigma_{sl} + \sigma_l \cos\ \theta \quad (3)$$

Substituting equations (1) and (2) into equation (3), we have

$$(1+\cos\ \theta)\sigma_l = 2\sqrt{\sigma_s^d\sigma_l^d} + 2\sqrt{\sigma_s^p\sigma_l^p} \quad (4)$$

The surface tension of a solid can be determined by utilizing just two liquids with known dispersive and polar surface tensions, exhibiting a significant polarity difference. Water and diiodomethane were chosen as test liquids for measuring truss surface tension because their dispersive and polar surface tensions are known.

$$(1+\cos\ \theta_W)\sigma_{lW} = 2\sqrt{\sigma_s^d\sigma_{lW}^d} + 2\sqrt{\sigma_s^p\sigma_{lW}^p} \quad (5)$$

$$(1+\cos\ \theta_D)\sigma_{lD} = 2\sqrt{\sigma_s^d\sigma_{lD}^d} + 2\sqrt{\sigma_s^p\sigma_{lD}^p} \quad (6)$$

where l_W represent water, l_D represent diiodomethane, and s represent the reactor truss materials. $\cos\ \theta_W$ represent the contact angle of water-air-solid truss materials $\text{and}\ \cos\ \theta_D$ represent the contact angle of diiodomethane-air-solid truss materials. Use equation 5 and 6 to solve for $\sigma_s^d\ and\ \sigma_s^p$ to calculate σs.

Supplementary Table 1. Surface tension data of water and diiodomethane at 20°C

Liquid	σ_l (mN/m)	σ_l^d (mN/m)	σ_l^p (mN/m)
l_W	72.8	21.8	51.0
l_D	50.8	49.5	1.3

Calculate the surface tension of the truss material.

$$\sigma_s = \sigma_s^d + \sigma_s^p = 62.46\ \text{mN/m}$$

Interfacial tension of water-truss material

$$\sigma_{\mathrm{sl}W} = \sigma_{\mathrm{s}} - \sigma_{lW}\cos\theta_W = 1.54\ \mathrm{mN/m}$$

$$\cos\ \theta_W = 33.2°$$

Interfacial tension of cyclohexane-truss material

$$\sigma_{\mathrm{l}C} = 26.33\ \mathrm{mN/m},\ \ \cos\ \theta_C = 0°$$

$$\sigma_{\mathrm{sl}C} = \sigma_{\mathrm{s}} - \sigma_{lC}\cos\theta_C = 36.13\ \mathrm{mN/m}$$

where l_C represent cyclohexane and $\cos\ \theta_C$ represent the contact angle of cyclohexane-air-solid truss materials.

Interfacial tension of water-cyclohexane

$$\sigma_{lWlC} = 44.35\ \mathrm{mN/m}$$

The three-phase contact angle is obtained according to the three-phase Young's equation of water-cyclohexane-truss material.

$$\sigma_{\mathrm{sl}W} = \sigma_{slC} - \sigma_{lWlC}\cos\theta$$

$$\theta = 38.7°$$

where $\cos\theta$ represent the contact angle of water-cyclohexane-solid truss material.

Supplementary Table 2. Description of interfacial tension

Interfacial tension	$\boldsymbol{\sigma_{lW}}$	$\boldsymbol{\sigma_{lD}}$	$\boldsymbol{\sigma_{s}}$	$\boldsymbol{\sigma_{lC}}$
Description	gas-liquid	gas-liquid	gas-solid	gas-liquid
Value (mN/m)	72.8	50.8	62.46	26.33
Source	measured	measured	calculated	measured
Interfacial tension	$\boldsymbol{\sigma_{lWlC}}$	$\boldsymbol{\sigma_{slC}}$	$\boldsymbol{\sigma_{slW}}$	
Description	liquid-liquid	solid-liquid	solid-liquid	
Value (mN/m)	44.35	36.13	1.54	
Source	measured	calculated	calculated	

Where *measured* denotes that it is derived from direct measurements or derived entirely from measured data. *Calculated* denotes that part of the data is derived from the literature and combined with measured data.

Supplementary Table 3. Description of inter contact angle

Contact angle	$\boldsymbol{\theta}$	$\mathbf{cos}\ \boldsymbol{\theta_W}$	$\mathbf{cos}\ \boldsymbol{\theta_C}$	$\mathbf{cos}\ \boldsymbol{\theta_D}$
Description	liquid-liquid-solid	gas-liquid-solid	gas-liquid-solid	gas-liquid-solid
Value (°)	38.7	33.2	0	136.2
Source	calculated	measured	measured	measured

Where *measured* denotes that it is derived from direct measurements or derived entirely from measured data. *Calculated* denotes that part of the data is derived from the literature and combined with measured data. The contact angle is given directly by a commercial contact angle system.

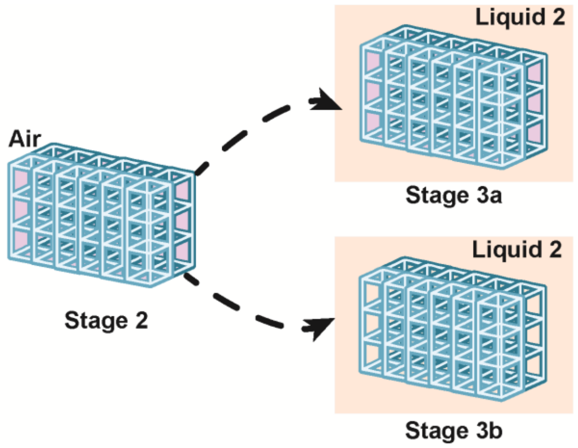

Supplementary Figure 4. Two possible states of the cellular reactor during stage 2 to stage 3

Two possibilities exist for moving the cellular reactor holding Liquid 1 into Liquid 2. If the cell truss exhibits a stronger affinity for the inner-phase liquid than the outer-phase liquid, Liquid 1 remains in the cellular reactor (holding, 3a). Conversely, it is replaced by Liquid 2 (leaking, 3b). The stability of the cellular reactor to hold liquid depends on whether the energy of the holding state (phase 3a) is lower than that of the leaking state (phase 3b), i.e., $E_{3a} < E_{3b}$.

$$E_{3a} = A_{SL2}\sigma_{2S} + A_{SL1}\sigma_{1S} + A_{LL}\sigma_{12} \tag{7}$$

$$E_{3b} = A_s\sigma_{2S} \tag{8}$$

The total contact area of the truss is the sum of the contact areas of the truss with liquid 1 and liquid 2, respectively：

$$A_s = A_{SL1} + A_{SL2} \tag{9}$$

Joining equations 7, 8, and 9, the solution is：

$$A_{SL1}\sigma_{1S} + A_{LL}\sigma_{12} < A_{SL1}\sigma_{2S} \quad (10)$$

According to the three-phase contact angle formula：

$$\sigma_{2S} = \sigma_{1S} + \sigma_{2S}\cos\ \theta \quad (11)$$

Substituting Equation 11 into Equation 10, we have：

$$A_{LL} - A_{SL1}\cos\ \theta < 0 \quad (12)$$

where A_{LL} is the contact area of the liquids 1 and 2, A_{SL1} is the contact area of liquid 1 with solid 3, and $\cos\ \theta$ is the contact angle of the three phases of liquids 1 and 2 with the solid.

Supplementary Table 4. Parameters of cellular reactors in this study

Reactor	Structure	Location	L	D*	Blades	N	Resin
Reactor-A	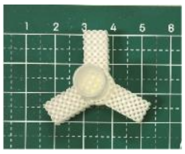	Fig. 3e	2 mm	0.33	3	498	CR
Reactor-B	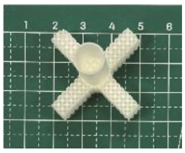	Fig. 3e	2 mm	0.33	4	568	CR
Reactor-C	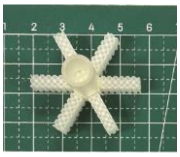	Fig. 3e	2 mm	0.33	6	708	CR
Reactor-D	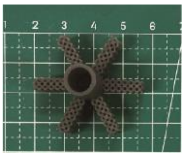		2 mm	0.33	6	708	TR
Reactor-E	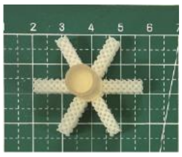		2 mm	0.33	6	708	HR
Reactor-F	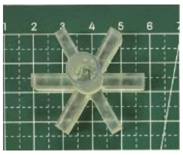		-	-	6	-	CR
Reactor-G	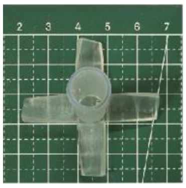		-	-	-	-	CR
Reactor-H	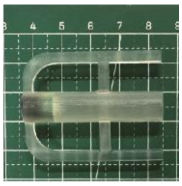		-	-	-	-	CR

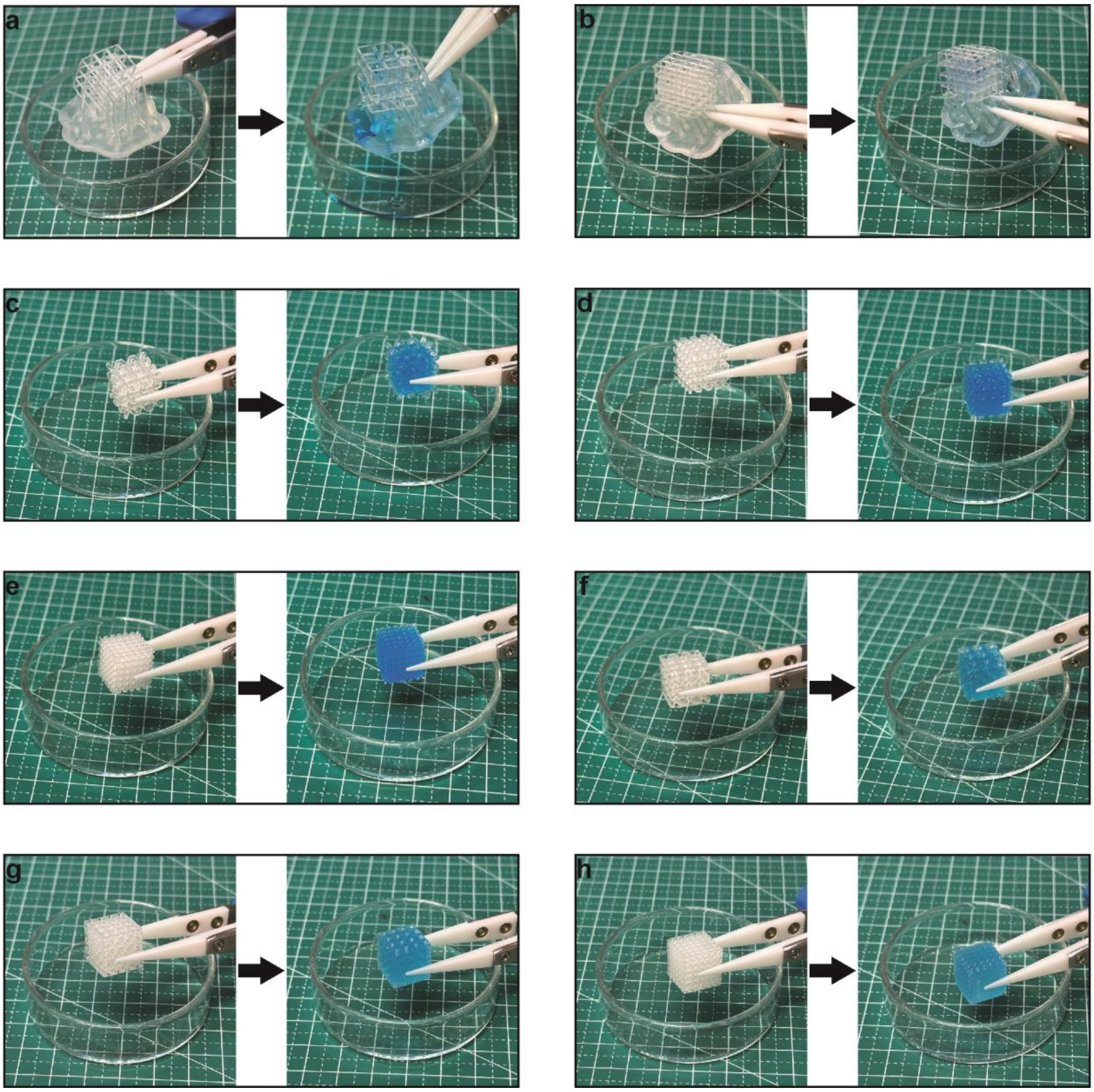

Supplementary Figure 5. Liquid state demonstration of different cellular reactors

Supplementary Figure 5 demonstrates the liquid retention effect. For cells S5-a&b, since they do not satisfy the formula $(\pi \frac{\lambda}{n} \cos\theta + 1)^{-1} < D^* < 1$, their operating points in Figure 3d, at n=2,3d, are located above the y=x line, and for n=1, very close to the y=x line, resulting in liquid leakage within the cells. However, for cells S5-c-h, the operating points are below the y=x line, effectively retaining the liquid. In this case, the

liquid is water, dyed with methylene blue.

Supplementary Table 5. Parameters of cellular reactors

Sample	Cellular type	L	D*	Liquid state
S5-a	Cubic	4	0.2	Leaked
S5-b	Cubic	3	0.2	Leaked
S5-c	BCC	4	0.333	Held
S5-d	BCC	3	0.333	Held
S5-e	BCC	2	0.333	Held
S5-f	BCC+frame	4	0.333	Held
S5-g	BCC+frame	3	0.333	Held
S5-h	BCC+frame	2	0.333	Held

Supplementary Table 6. Catalytic performance of different fractionated cellular reactors

Reactor	Time 1	Conversion rate 1	Time 2	Conversion rate 2
Reactor -A	60 min	15.4%	120 min	34.6%
Reactor -B	60 min	40.8%	120 min	70.7%
Reactor -C	60 min	50.8%	120 min	83.6%
Reactor -F	60 min	2.48%	90 min	4.86%

Dihydropyran (12.5 mmol) and benzyl alcohol (12.5 mmol, 1.0 equiv) were combined in 50 mL of cyclohexane. A 0.1 M H_2SO_4 solution was adsorbed into the cellular reactor and inserted into the organic phase, stirring at 50 rpm. The reactions were conducted in custom-made three-neck flasks heated to 60°C. Three different fractions of the cellular reactor were utilized. For Reactor-F, an H_2SO_4 solution equivalent to the volume of liquid held by the cellular reactor was added to the flask, and a solid stirring paddle without cell structure was used to stir the reaction at the same rpm.

The necessity of the cell structure design in the cellular reactor is evident from the use of solid stirring paddles. This design significantly enhances the reaction area and boosts the reaction rate while accurately containing the corrosive component (sulfuric acid) within the cellular reactor to prevent reactor corrosion.

Molecular dynamics simulation

The density functional theory (DFT) calculations were carried out with the VASP code. The Perdew–Burke–Ernzerhof (PBE) functional within generalized gradient approximation (GGA) was used to process the exchange-correlation. At the same time, the projected augmented wave pseudopotential (PAW) was applied with a kinetic energy cut-off of 500 eV, which was utilized to describe the expansion of the electronic eigenfunctions. The vacuum thickness was set to 15 Å to minimize interlayer interactions. The Brillouin-zone integration was sampled by a Γ-centered 5 × 5 × 1 Monkhorst–Pack k-point. All atomic positions were fully relaxed until energy and force reached a tolerance of 1×10^{-5} eV and 0.03 eV/Å, respectively. The dispersion-corrected DFT-D method was employed to consider the long-range interactions. We employed a timestep of 100,000 during the molecular dynamics simulation for the time integration. We considered the degrees of freedom of atoms in all directions and recorded the dynamical trajectories for subsequent analysis.

The Verlet algorithm is a commonly used numerical integration method for updating the positions and velocities of atoms in molecular dynamics simulations. It is based on Newton's second law and Taylor expansion, utilizing the current and previous positions to estimate the next position.

The basic idea of the Verlet algorithm is to update positions and velocities by calculating the atomic accelerations. The steps involved are as follows:

1. Initialization: Provide initial positions and velocities and compute the initial

accelerations.

2. Position update: Using the current positions, velocities, and accelerations, update the positions of atoms using the following equation:

$$r(t+\Delta t) = 2r(t) - r(t-\Delta t) + a(t)\Delta t^2$$

where $r(t)$ represents the current position, $r(t-\Delta t)$ represents the previous position, $a(t)$represents the current acceleration, and Δt is the time step.

3. Acceleration update: Calculate the new accelerations based on the updated positions:

$$a(t+\Delta t) = F(r(t+\Delta t))/m$$

where $F(r(t+\Delta t))$ represents the force calculated from the potential energy function, and m is the mass of the atom.

4. Velocity update: Update the velocities using the following equation:

$$v(t+\Delta t) = \frac{r(t+\Delta t) - r(t-\Delta t)}{2\Delta t}$$

Repeating these steps updates the positions and velocities of atoms at each time step. The Verlet algorithm is known for its numerical stability and ability to conserve total energy, making it widely used in molecular dynamics simulations.

Modeling of interfacial reactions. To better understand the physicochemical mechanisms involved in cellular reactors, we constructed a double membrane model akin to the interphase mass transfer to investigate the chemical reactions occurring at the interface.

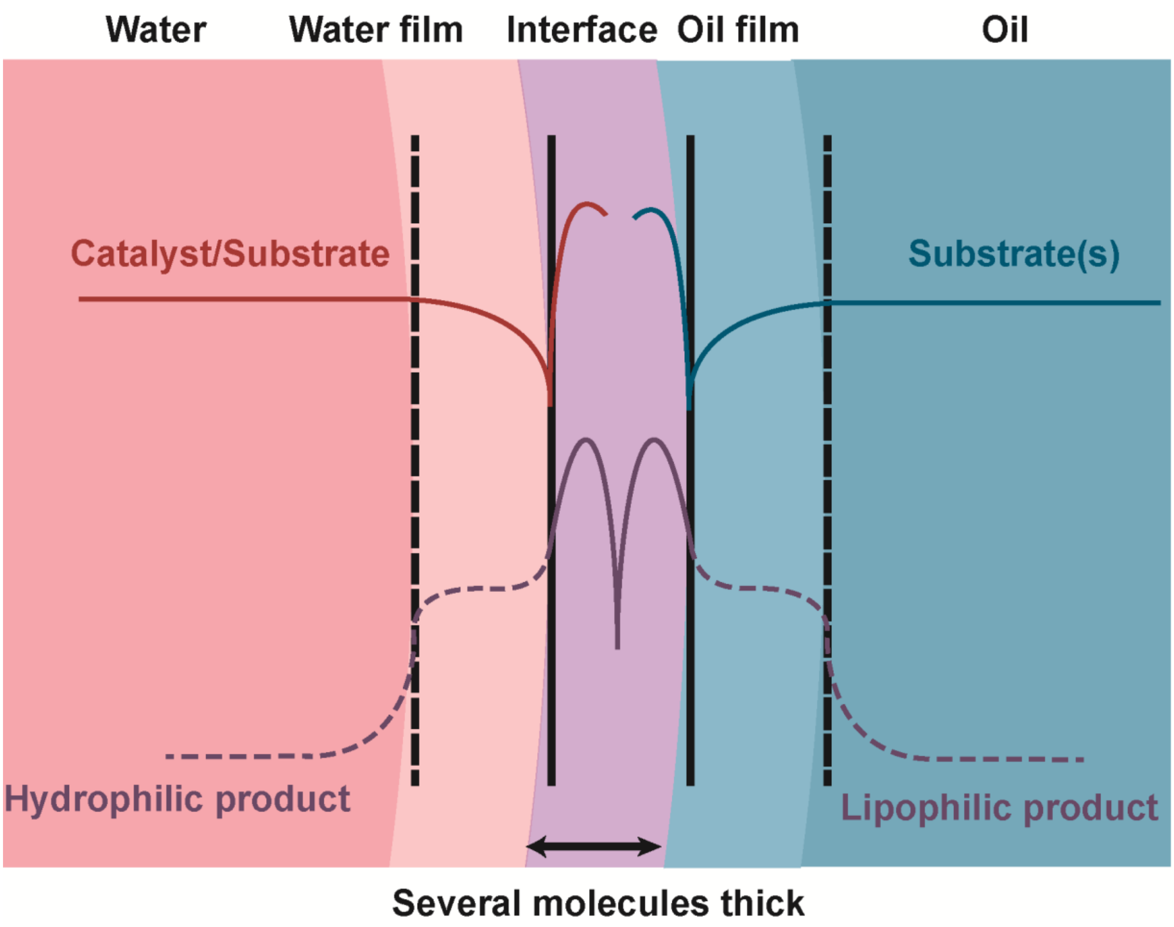

Supplementary Figure 6. illustrates a dual-membrane model for interface reactions.

The proposed interfacial reaction model encompasses the following key aspects:

(1). A stable phase interface exists between the two liquid phases. Solute molecules overcome the mass transfer resistance of their respective membranes to reach the phase interface.

(2). The solution composition within the bulk phase is homogeneous, while the substrate concentration at the phase interface is lower than that in the bulk phase.

(3). Substrate molecules encounter the catalyst at the interface, initiating the reaction process. Subsequently, substrate molecules are consumed in the reaction and

replenished by molecules from the bulk phase until equilibrium is achieved.

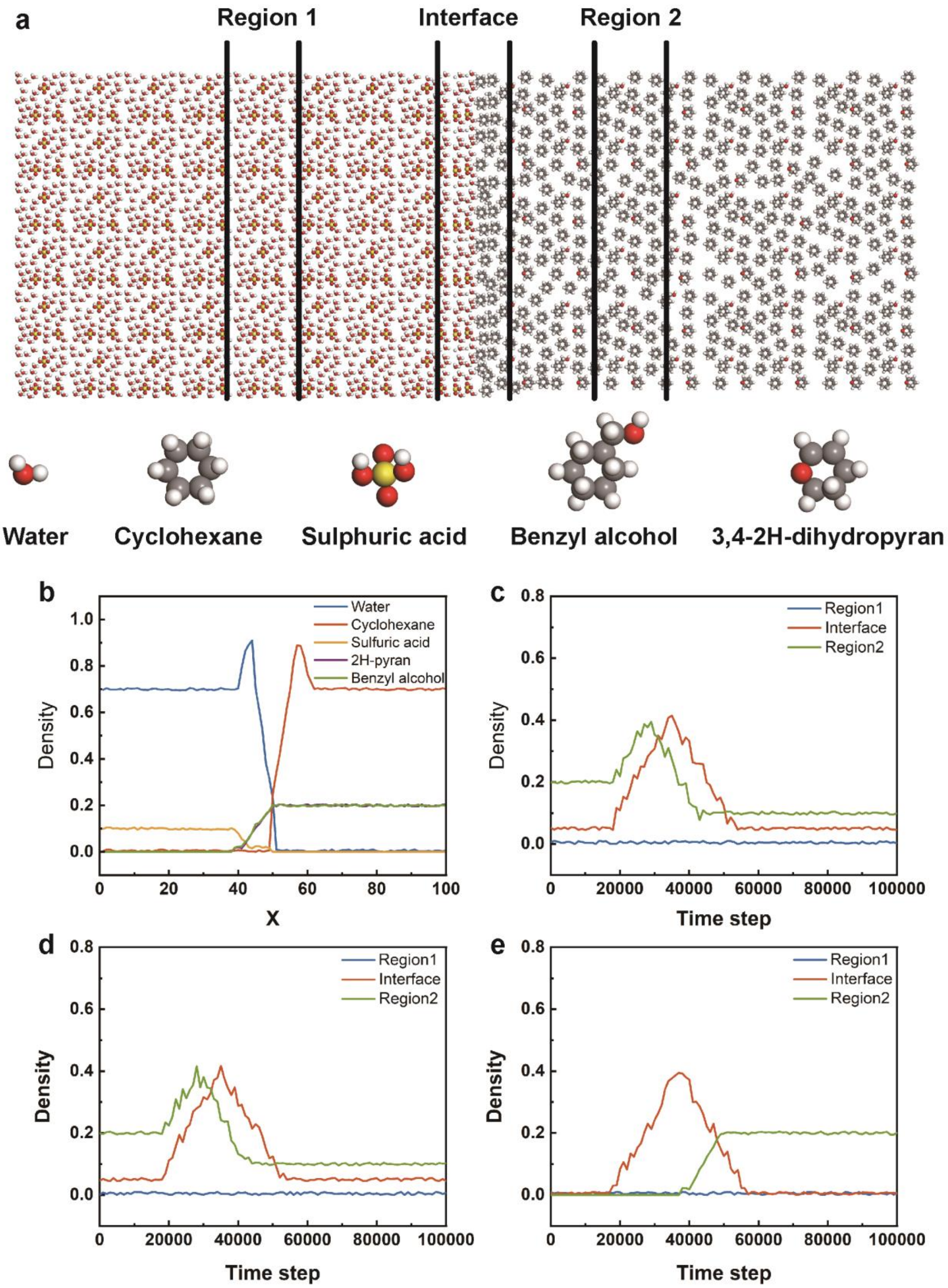

Supplementary Figure 7. Molecular Dynamics Simulation (a) Schematic diagram of the reaction system (b) Molecular density of each substance (Relative position) (c) Density distribution of dihydropyran (d) benzyl alcohol and (f) tetrahydropyran ether molecules (the molecular density is relative density)

(4). The product molecules dissolve in either the aqueous or oil phase (typically the oil phase) based on the difference in partition coefficients between these two phases.

To validate the accuracy of the reaction model, we conducted molecular simulations based on first principles calculations to examine the molecular distribution at the interface. The solvents used were water and cyclohexane for regions 1 and 2. The solutes consisted of sulphuric acid (region 1), dihydropyran, and benzyl alcohol (region 2) (Supplementary Fig. 7a). The water-oil interface was well-defined, situated at approximately 50 along the x-axis direction (Supplementary Fig.7b). Solute concentration in the bulk phase exhibited uniform distribution. As solute molecules approached the interface for the reaction, they traversed the corresponding water/oil film, decreasing concentration to about 20% of the bulk phase at the interface and nearly zero concentration in the other phase. Notably, substrate molecules showed enrichment upon reaching the interface (Supplementary Figs. 7c and d), leading to the appearance of product tetrahydropyran ether once they reached the minimum reactive concentration (Supplementary Fig. 7e). The chemical potential at the interface induced

diffusion of substrate molecules from the bulk phase, thereby increasing the local concentration. Likewise, product concentration escalated rapidly at the interface. The product was readily soluble in cyclohexane and had an almost negligible molecular density in water. These calculations demonstrate that our theoretical model aligns with the discoloration reaction phenomenon observed in previous experiments. The water-oil interface formed by the cellular reactor facilitates molecular enrichment and facilitates the reaction's progression. Moreover, the resulting products are primarily confined to the oil phase outside the reactor, impeding their diffusion into the water phase within the reactor, which is advantageous for subsequent separation.

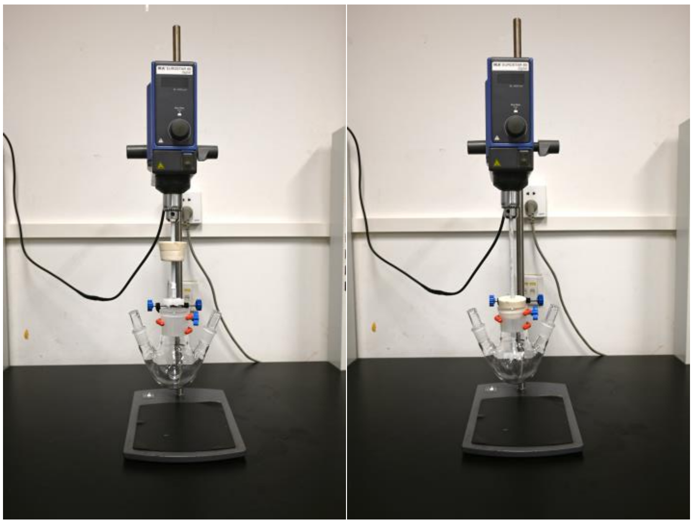

Supplementary Figure 8. Photograph of the reaction unit

GC-MS, UPLC-MS and NMR Data

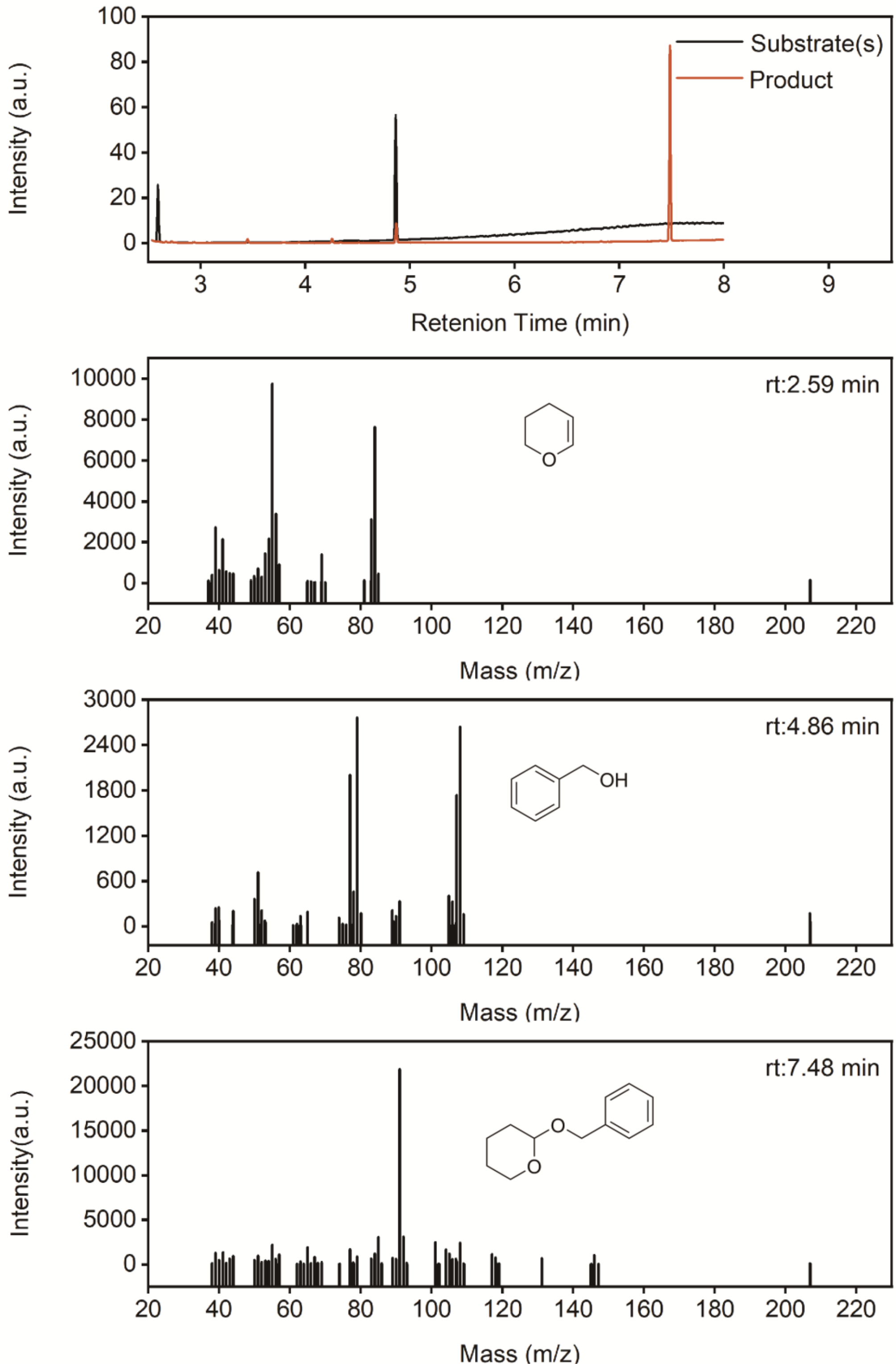

Supplementary Figure 9. GC-MS spectrum of entry 1

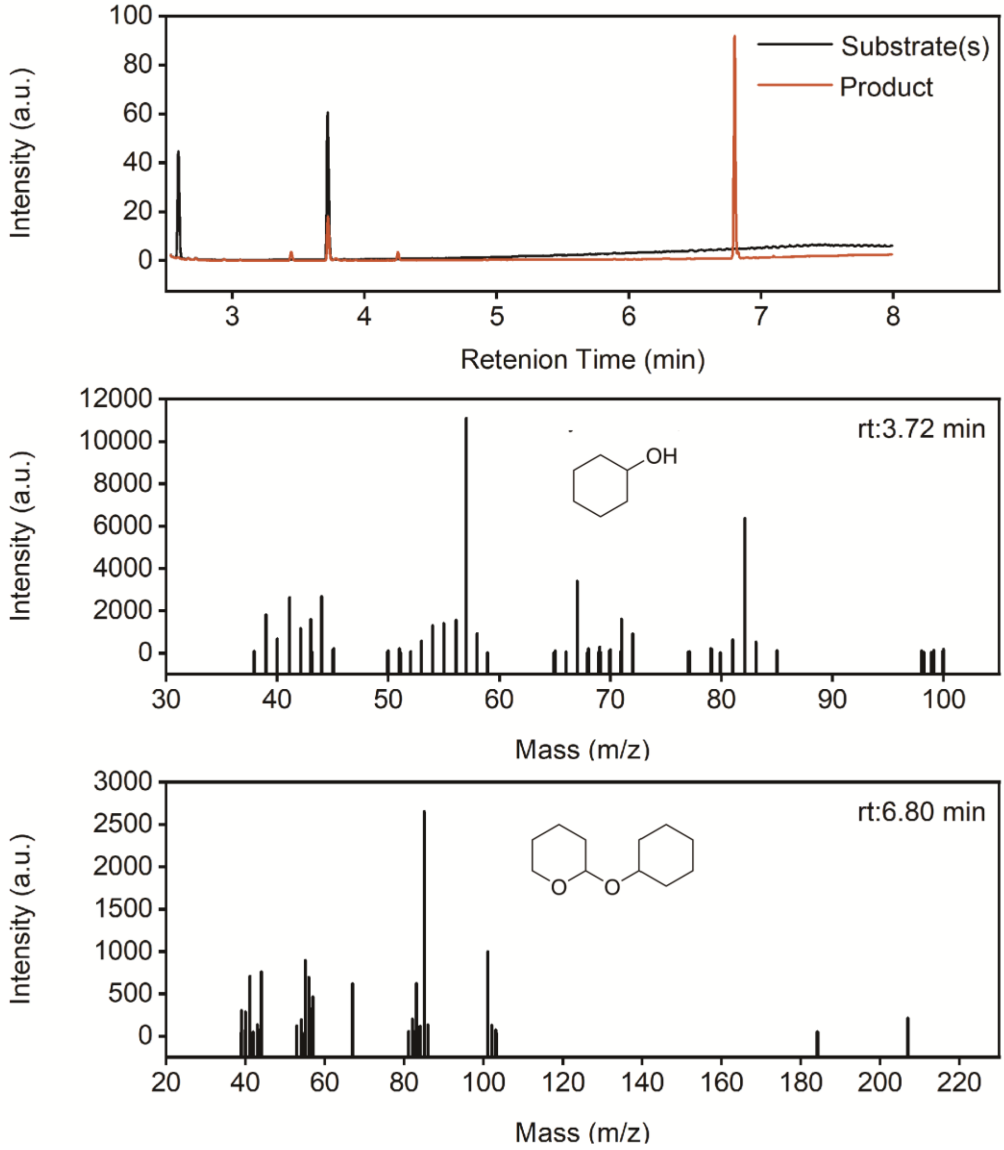

Supplementary Figure 10. GC-MS spectrum of entry 2

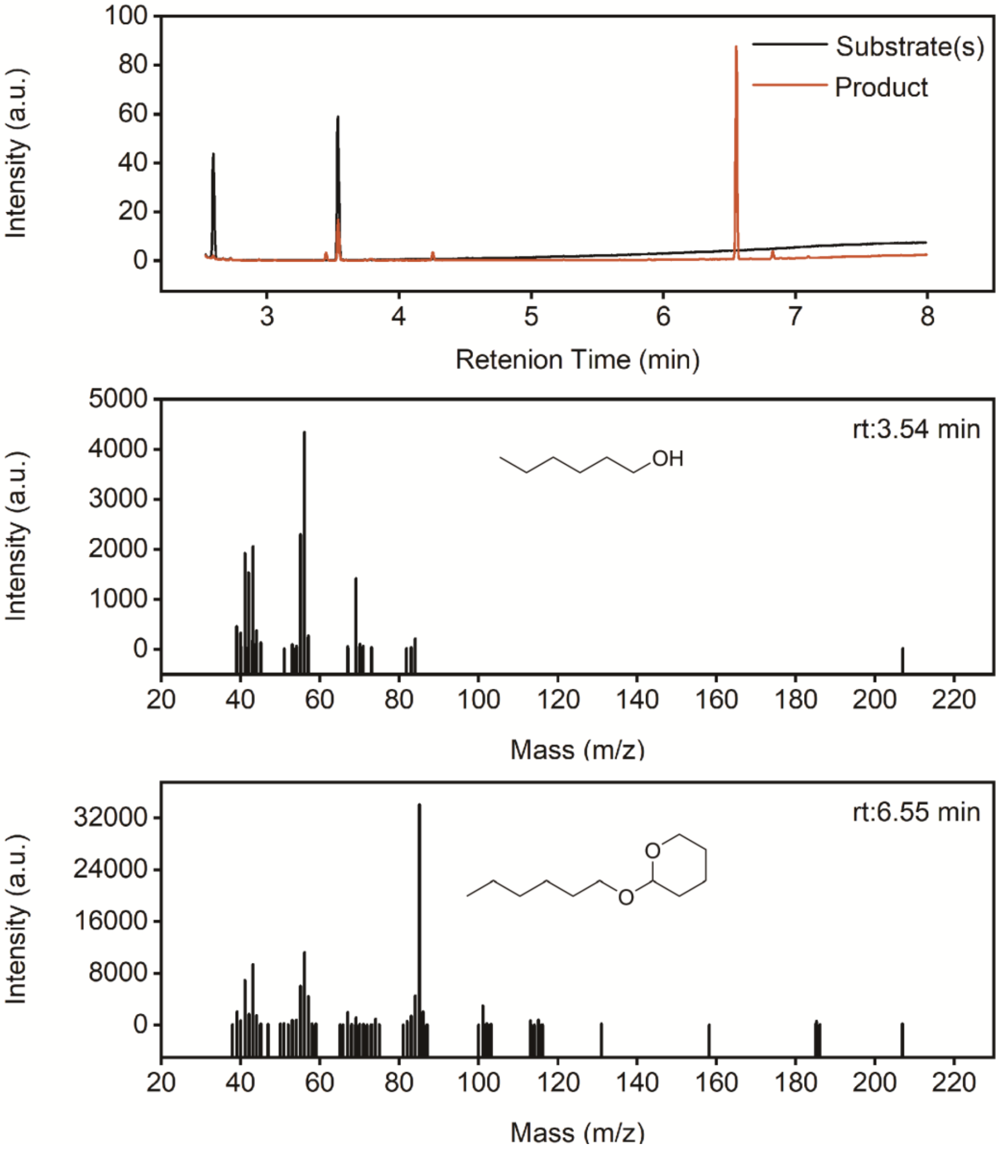

Supplementary Figure 11. GC-MS spectrum of entry 3

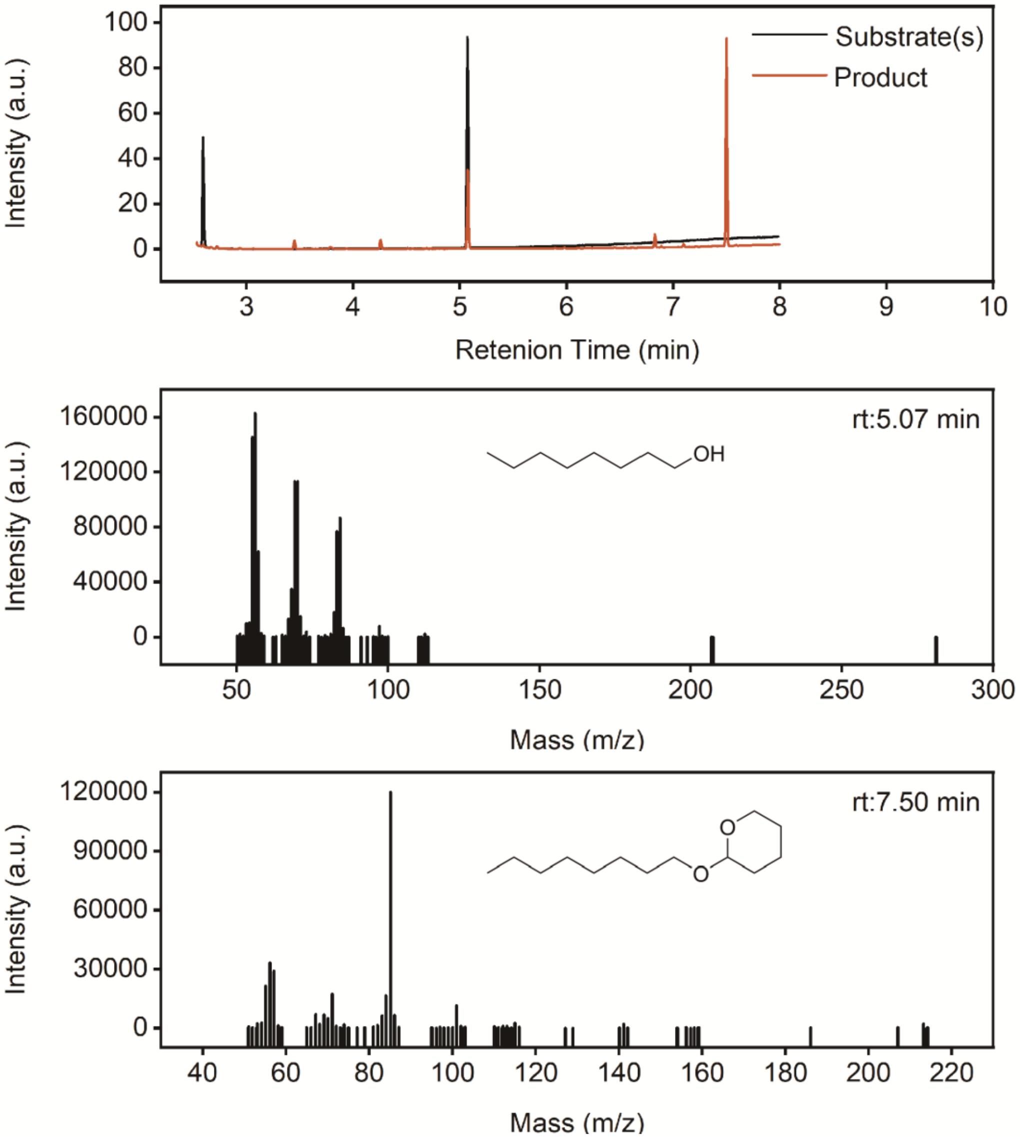

Supplementary Figure 12. GC-MS spectrum of entry 4

Supplementary Table 7. Blank control experiments for Knoevenagel condensation reaction

Time	Conversion rate
0	0
260 min	7.7%

A mixture of 2.5 mmol 4-chlorobenzaldehyde and 7.5 mmol malononitrile (3 eq.) was dissolved in 50 mL of ethyl acetate. For the control experiment, pure water was introduced into the cellular reactor as a blank catalyst and then inserted into the organic phase. The reaction took place in a custom-made three-necked flask and was catalyzed at 60 °C with stirring at 50 rpm. The reactor type is Reactor-C in Supplementary Table 4.

Blank control experiments were conducted to assess the requirement for K_2CO_3. After 260 minutes of reaction, the conversion of 4-chlorobenzaldehyde was approximately 8%, significantly lower than the conversion observed in the presence of K_2CO_3. This result demonstrates the essential nature of using K_2CO_3 in the reaction.

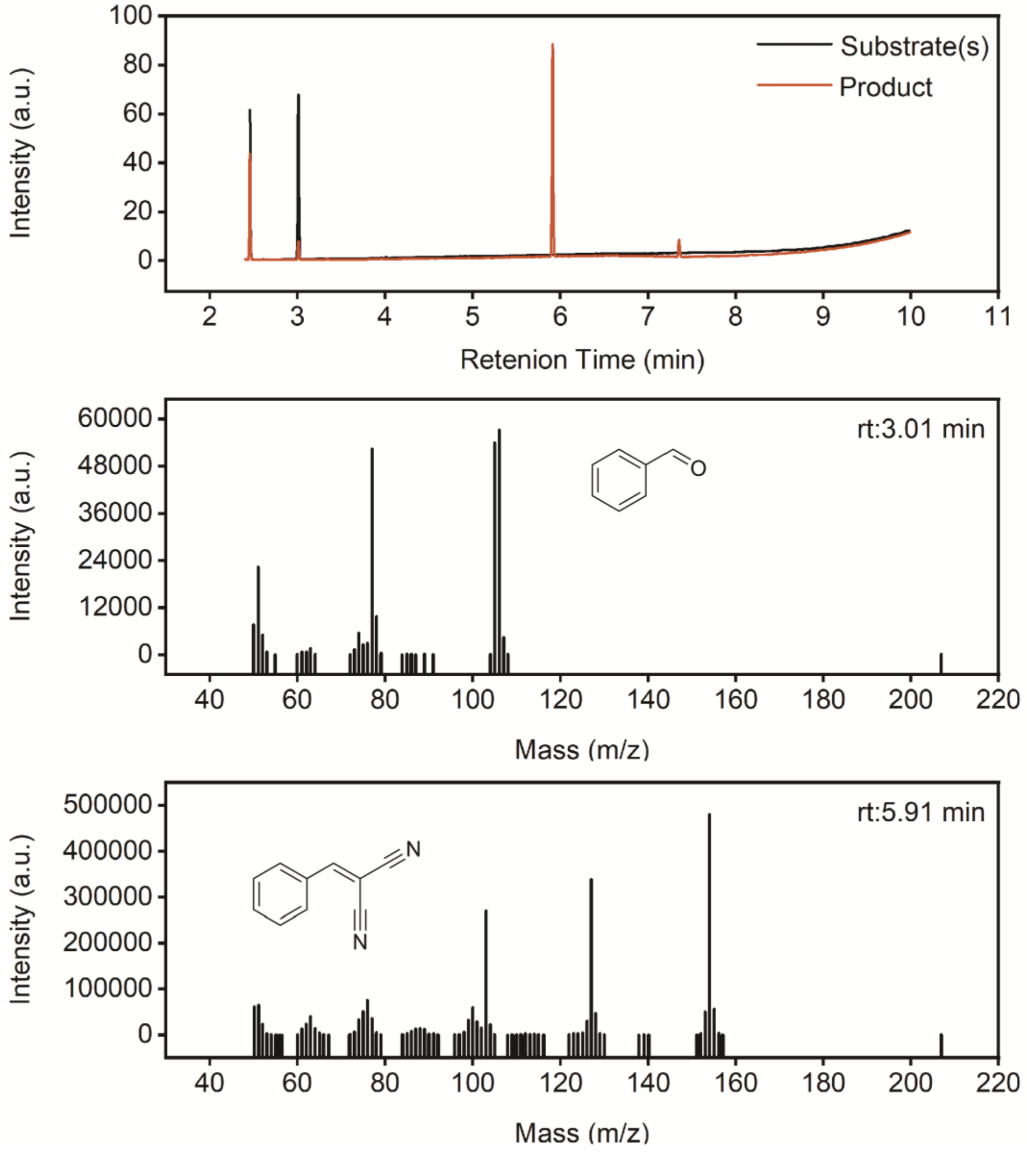

Supplementary Figure 13. GC-MS spectrum of entry 5

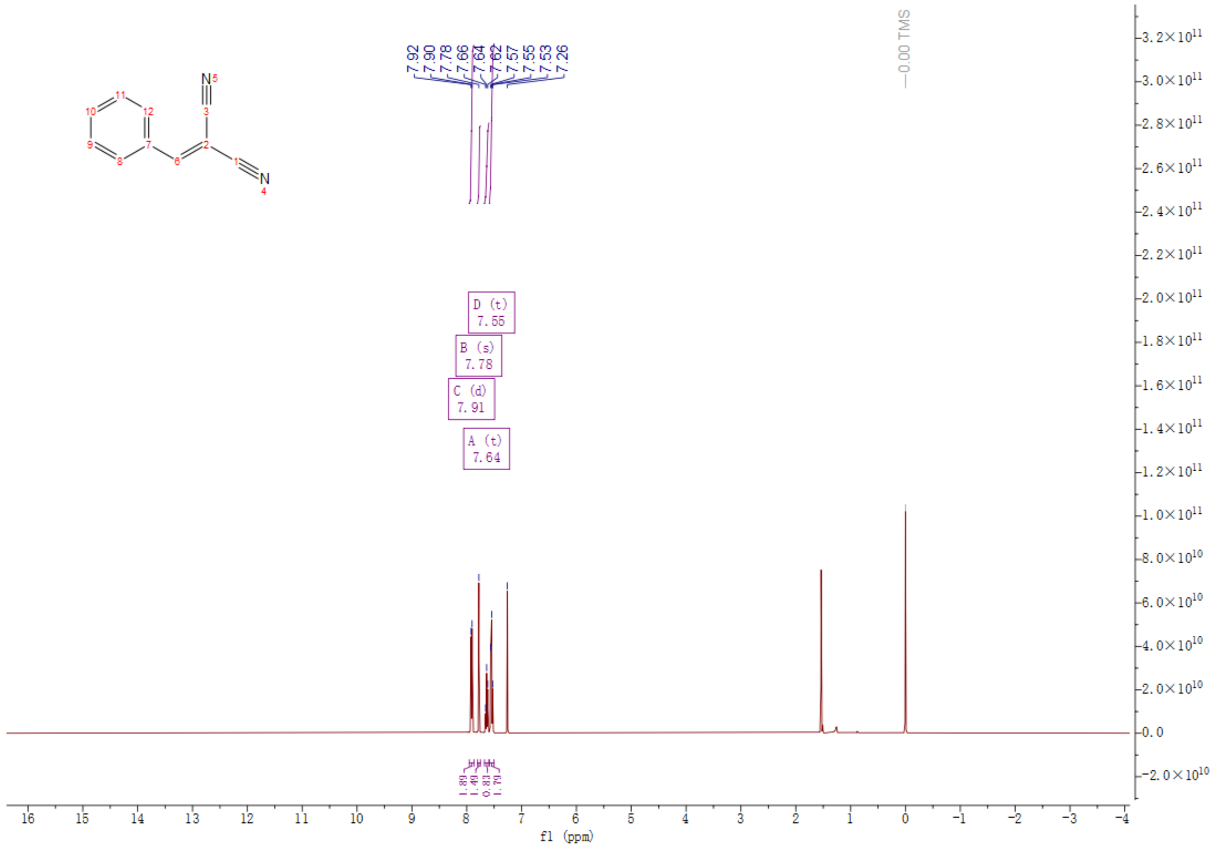

Supplementary Figure 14. NMR spectrum of Benzylidenemalonontrle

1H NMR (400 MHz, CDCl3) δ 7.91 (d, J = 7.4 Hz, 2H), 7.78 (s, 1H), 7.64 (t, J = 7.4 Hz, 1H), 7.55 (t, J = 7.6 Hz, 2H).

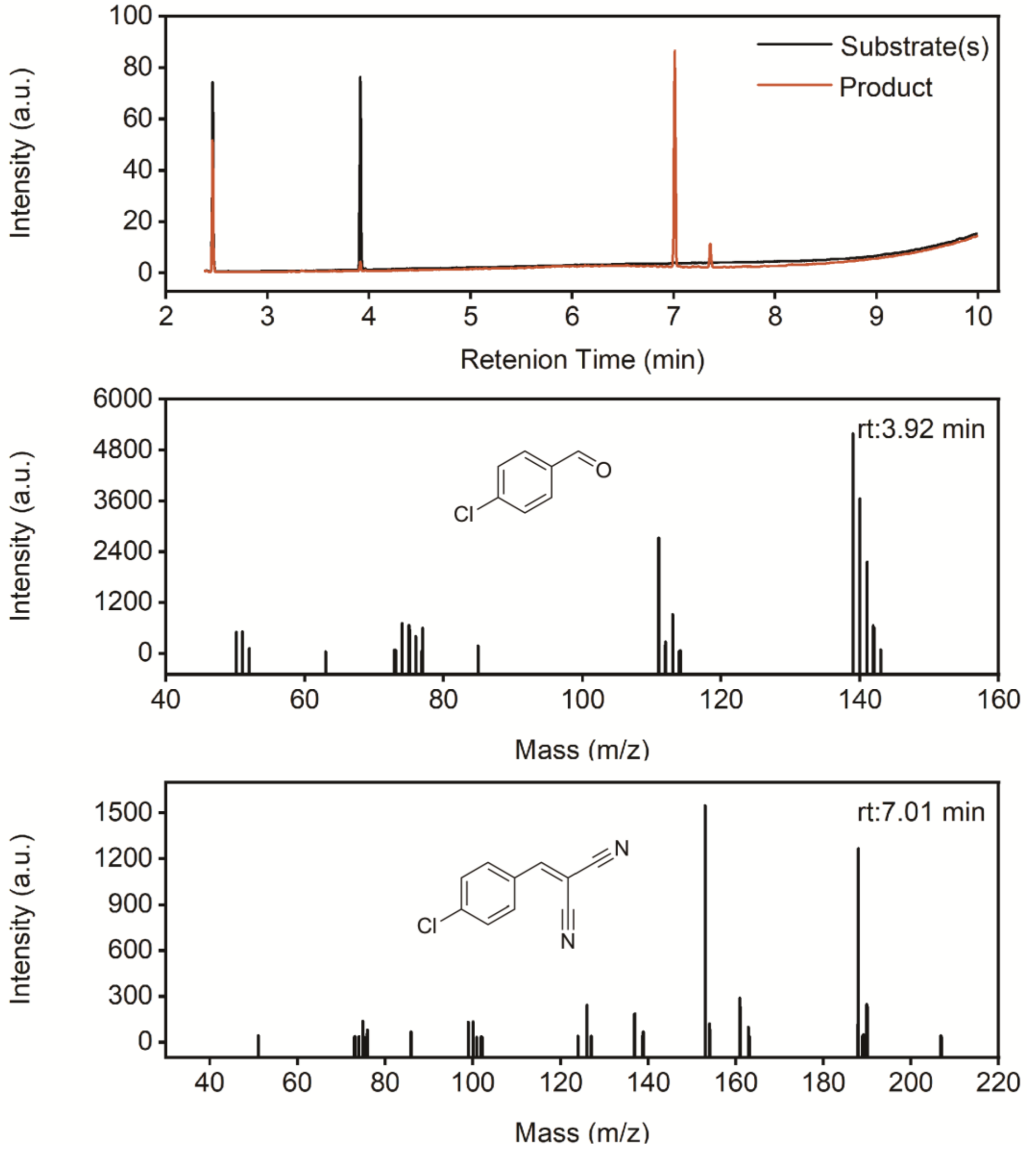

Supplementary Figure 15. GC-MS spectrum of entry 6

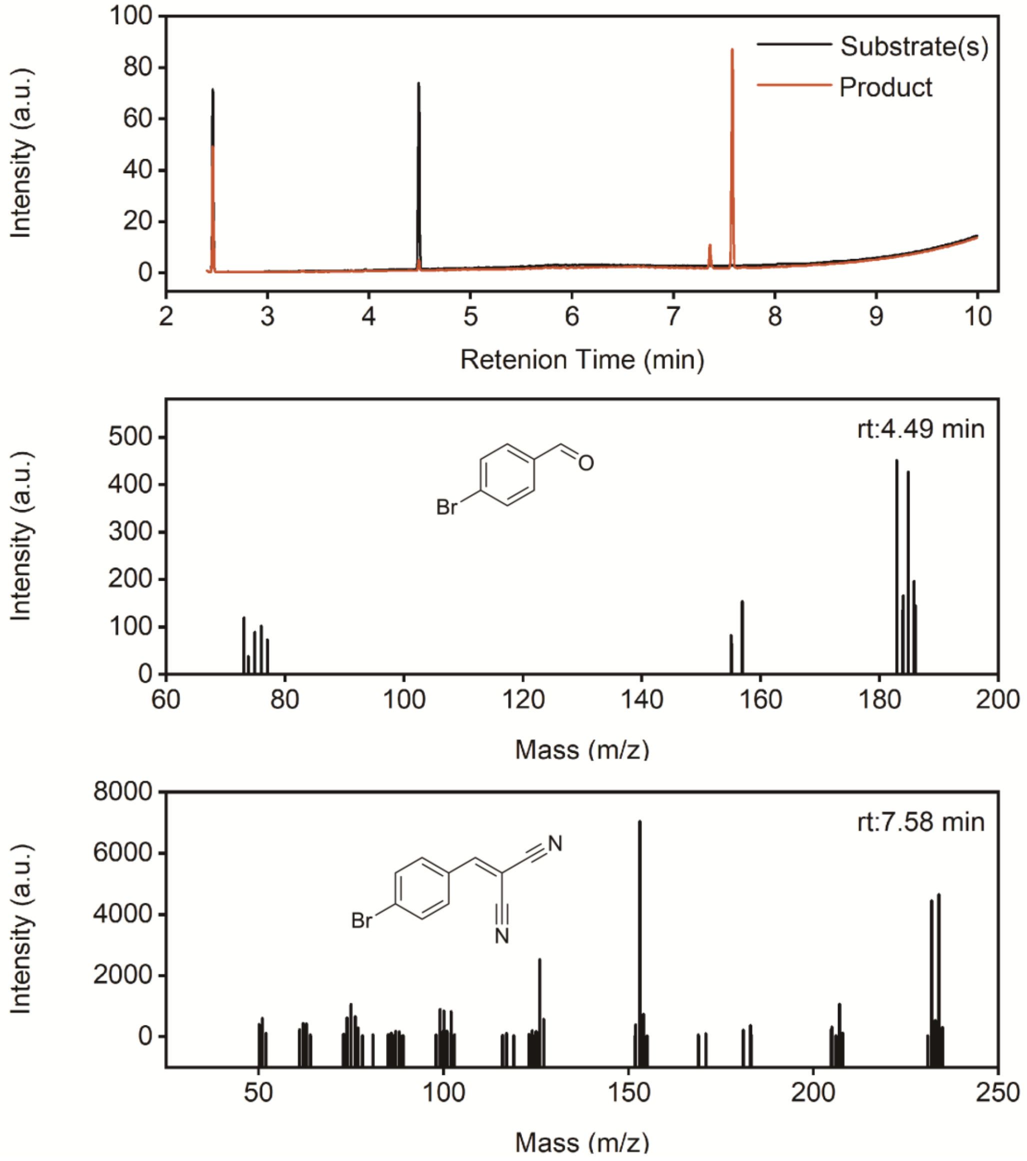

Supplementary Figure 16. GC-MS spectrum of entry 7

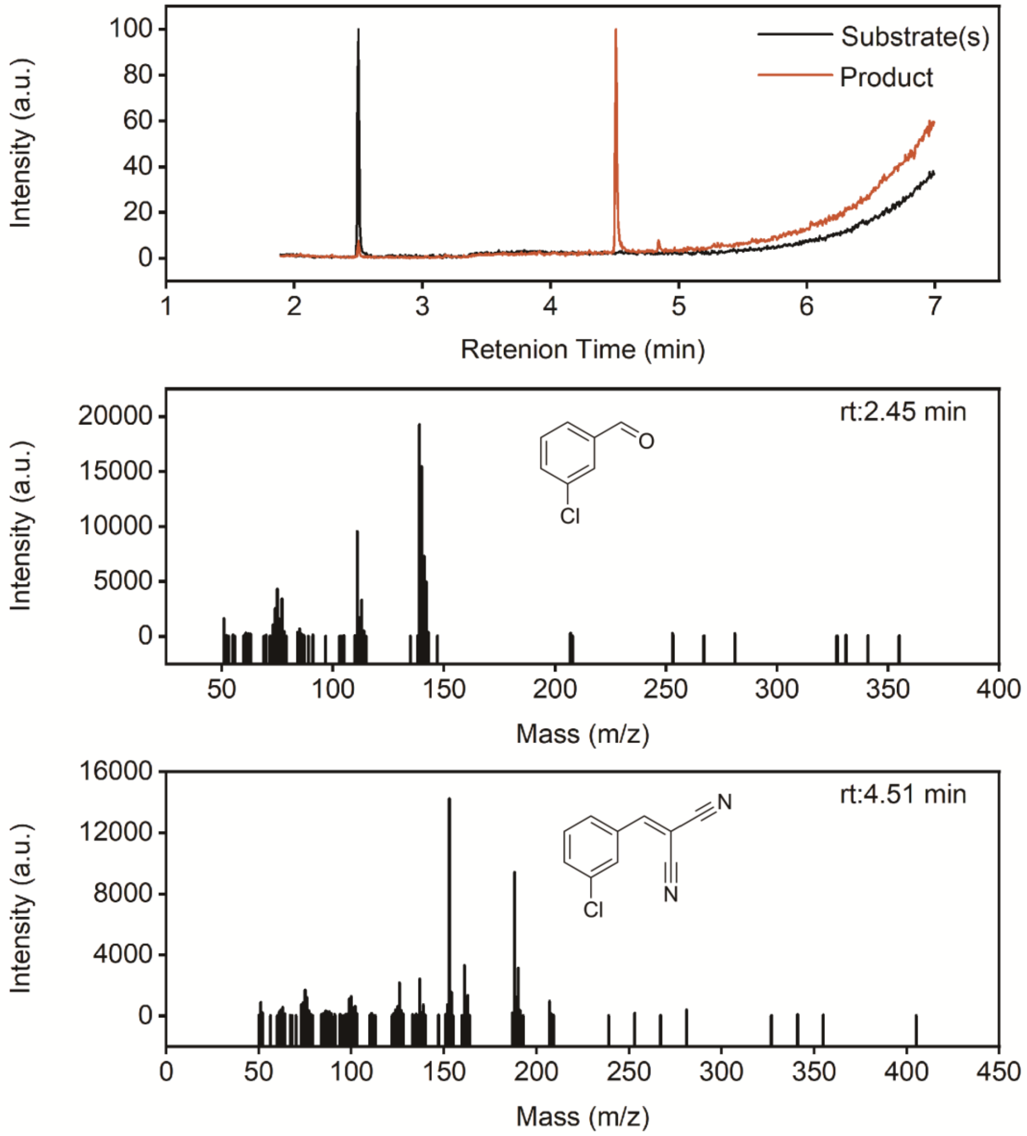

Supplementary Figure 17. GC-MS spectrum of entry 8

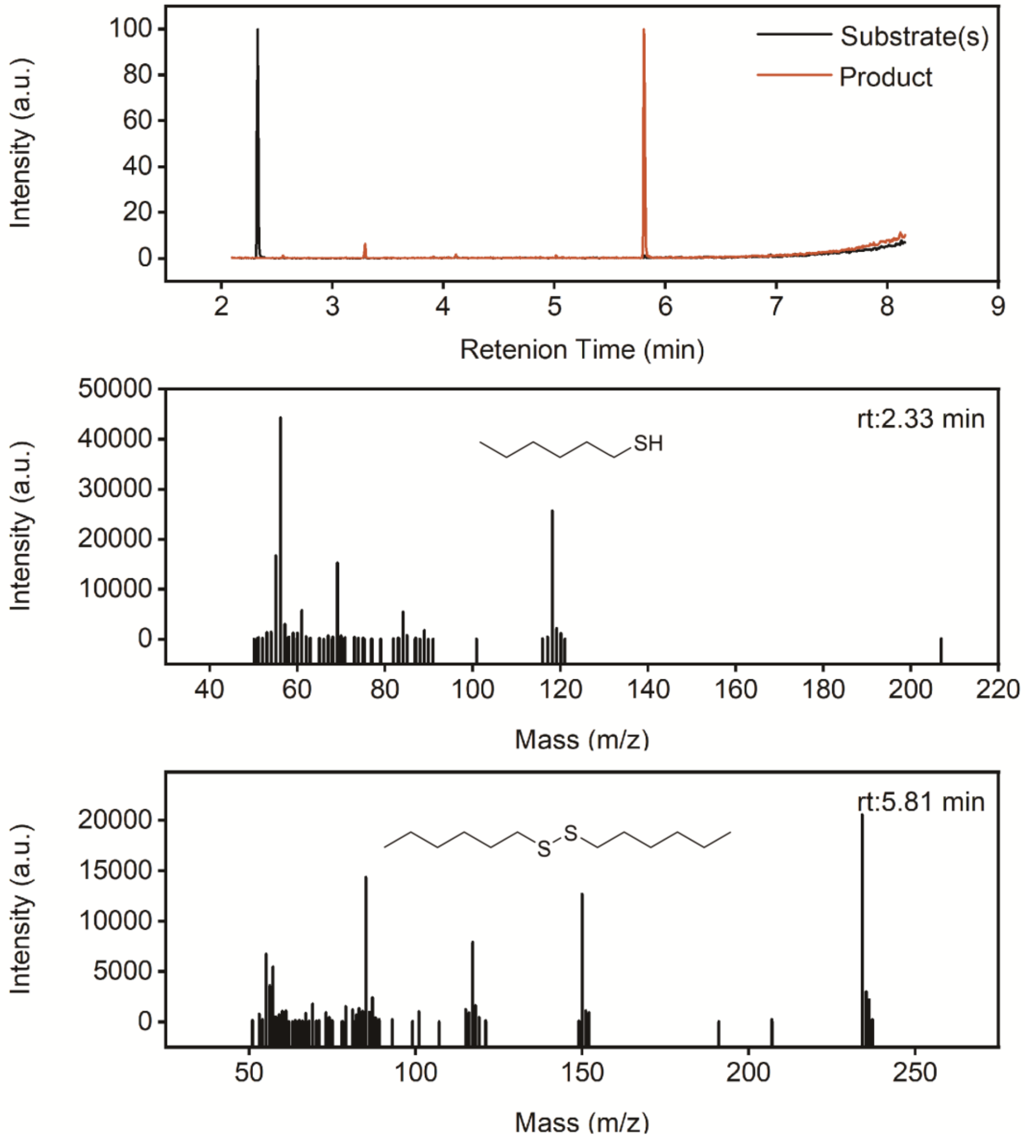

Supplementary Figure 18. GC-MS spectrum of entry 9

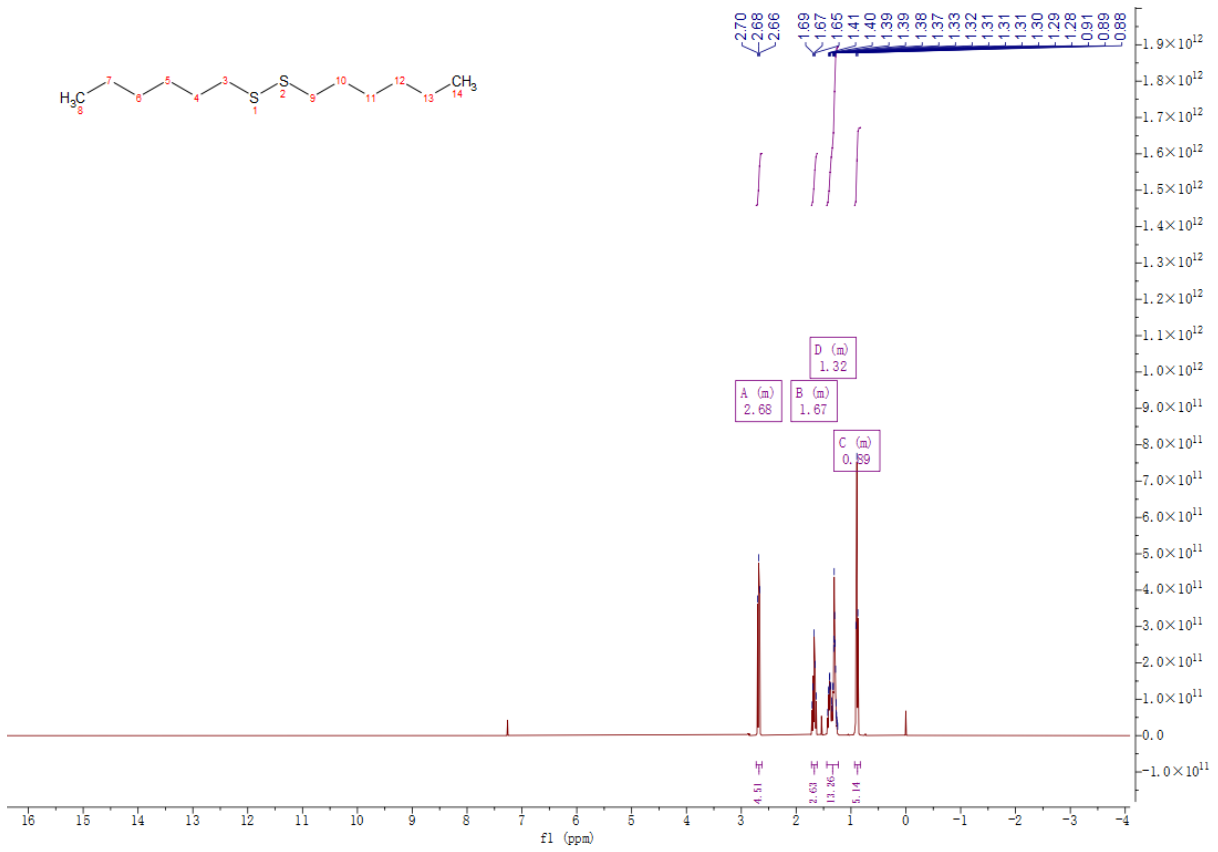

Supplementary Figure 19. NMR spectrum of Di-n-hexyl-disulfide

1H NMR (400 MHz, CDCl3) δ 2.73 – 2.62 (m, 5H), 1.72 – 1.62 (m, 3H), 1.44 – 1.23 (m, 13H), 0.93 – 0.83 (m, 5H).

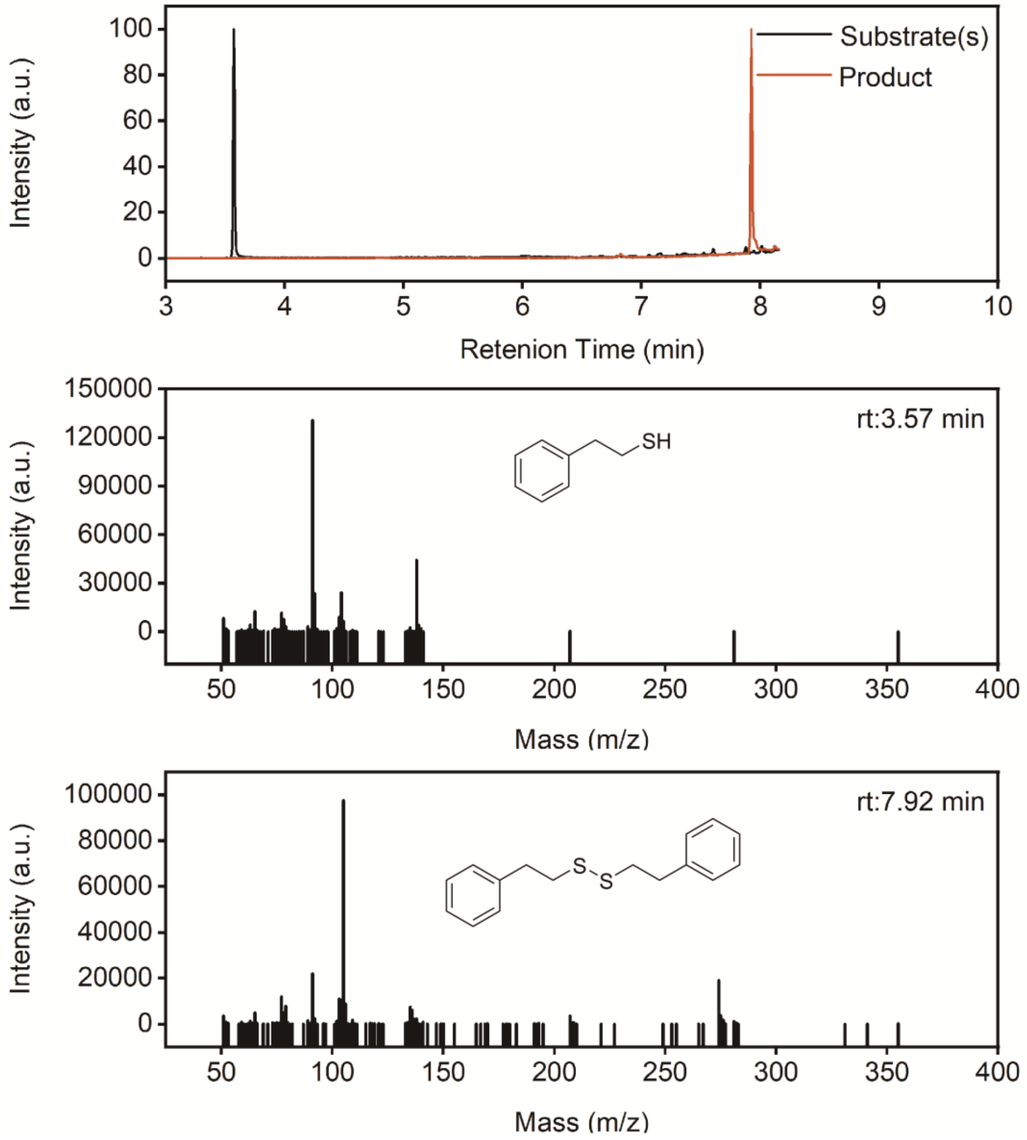

Supplementary Figure 20. GC-MS spectrum of entry 10

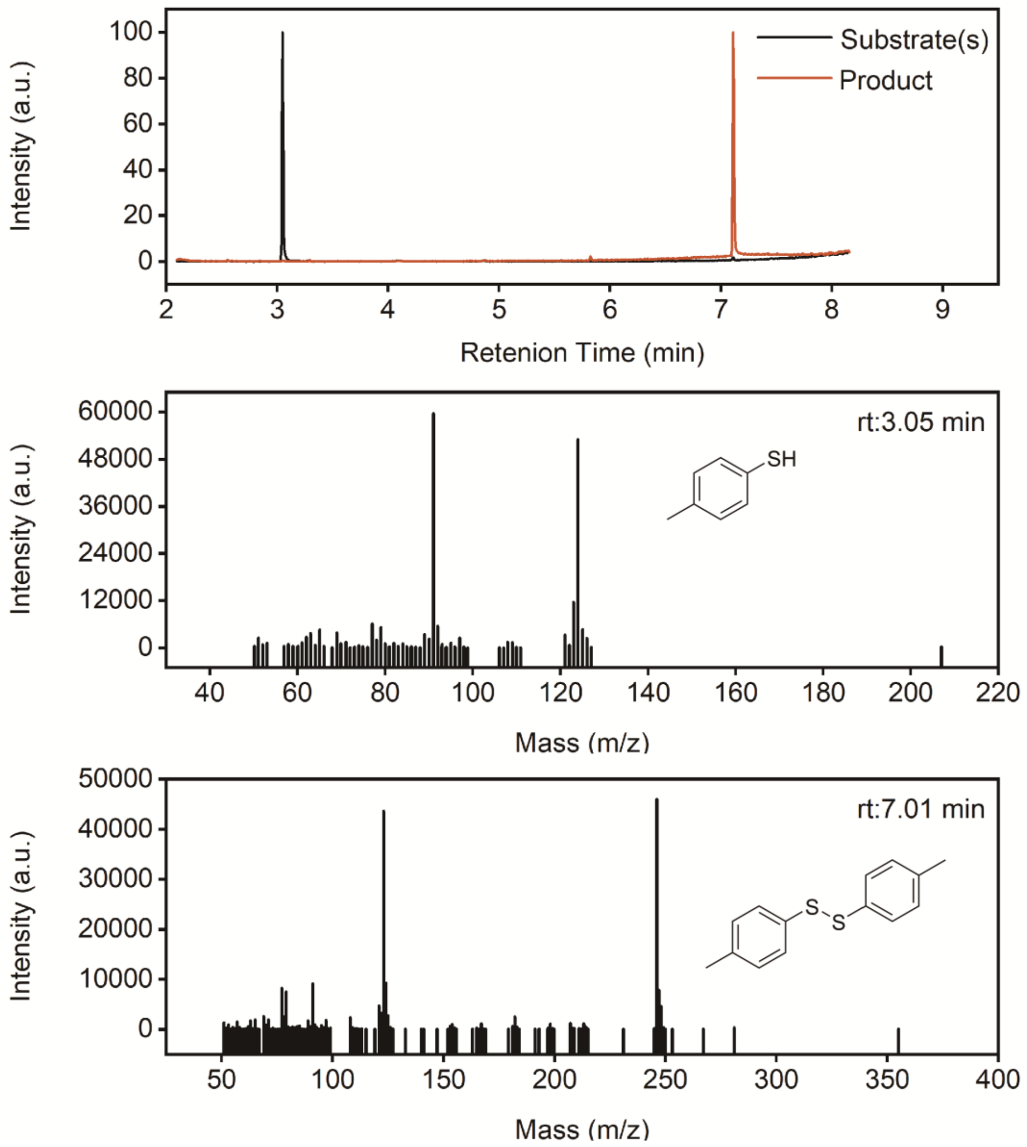

Supplementary Figure 21. GC-MS spectrum of entry 11

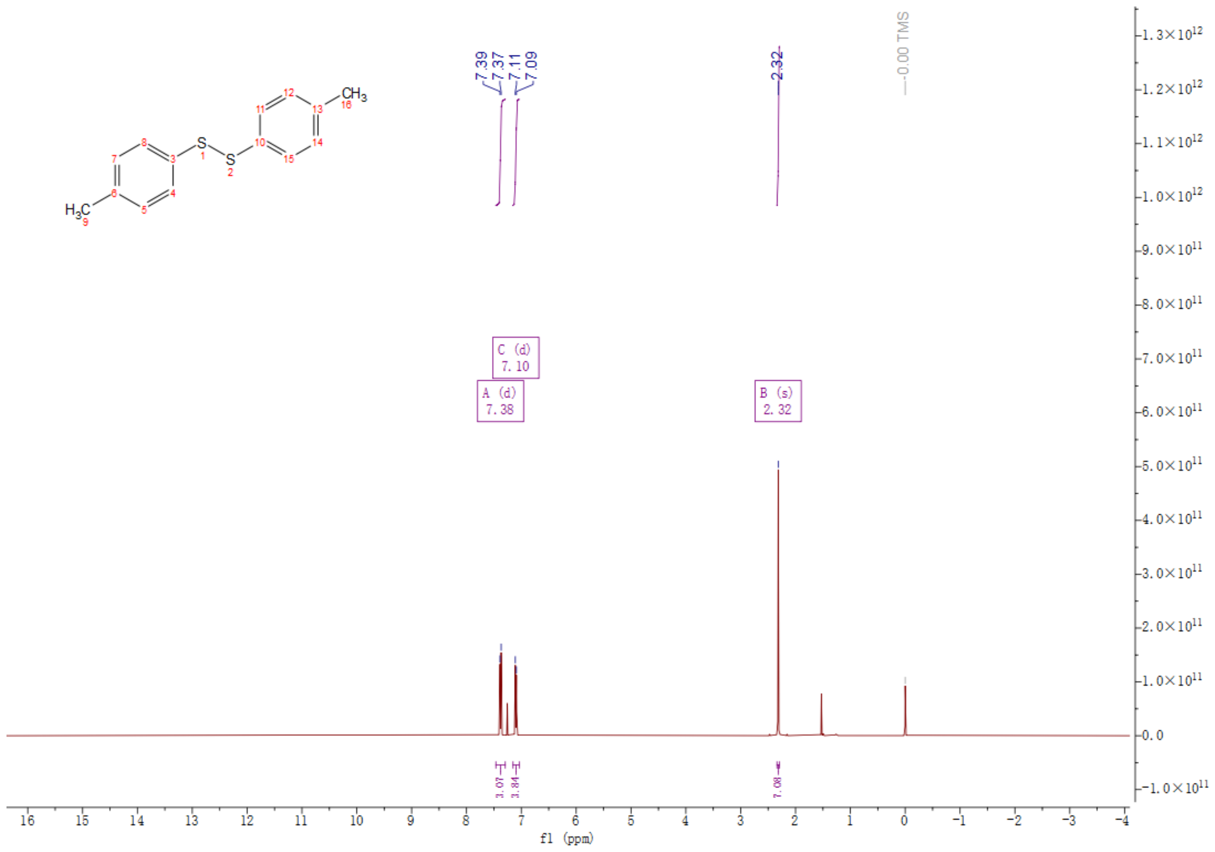

Supplementary Figure 22. NMR spectrum of 4,4'-dimethyldiphenyldisulphide

1H NMR (400 MHz, CDCl3) δ 7.38 (d, J = 8.3 Hz, 3H), 7.10 (d, J = 8.1 Hz, 4H), 2.32 (s, 7H).

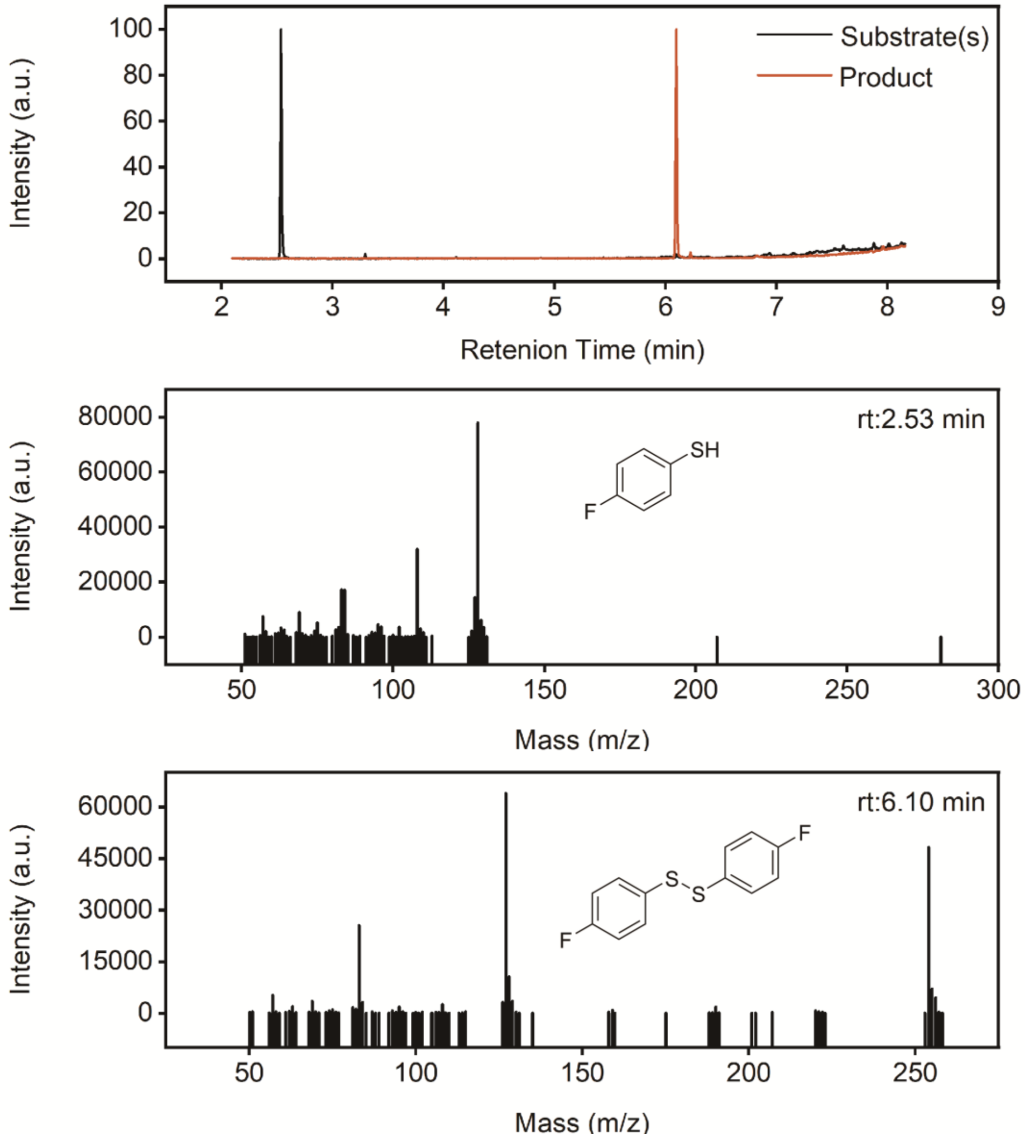

Supplementary Figure 23. GC-MS spectrum of entry 12

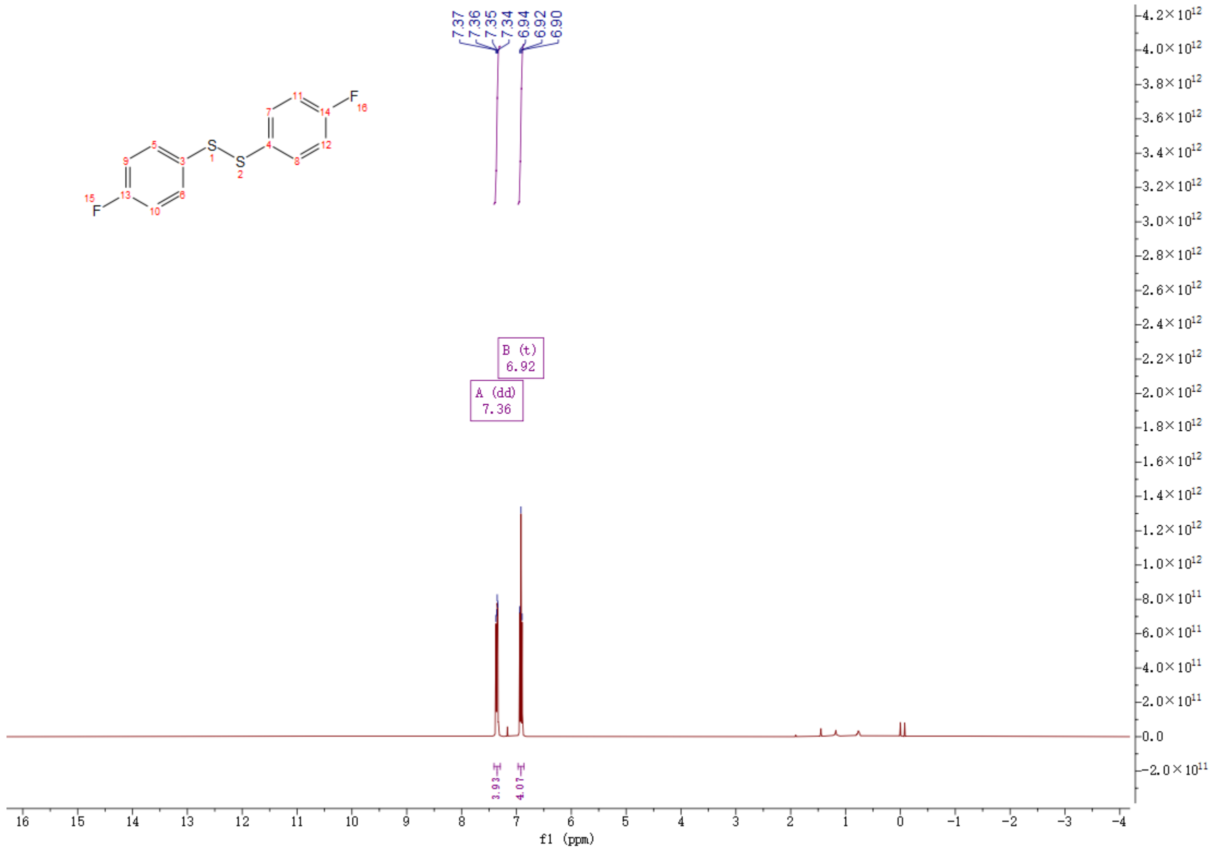

Supplementary Figure 24. NMR spectrum of Di-4-fluorophenyl sulfide

1H NMR (400 MHz, CDCl3) δ 7.36 (dd, J = 8.9, 5.1 Hz, 4H), 6.92 (t, J = 8.7 Hz, 4H).

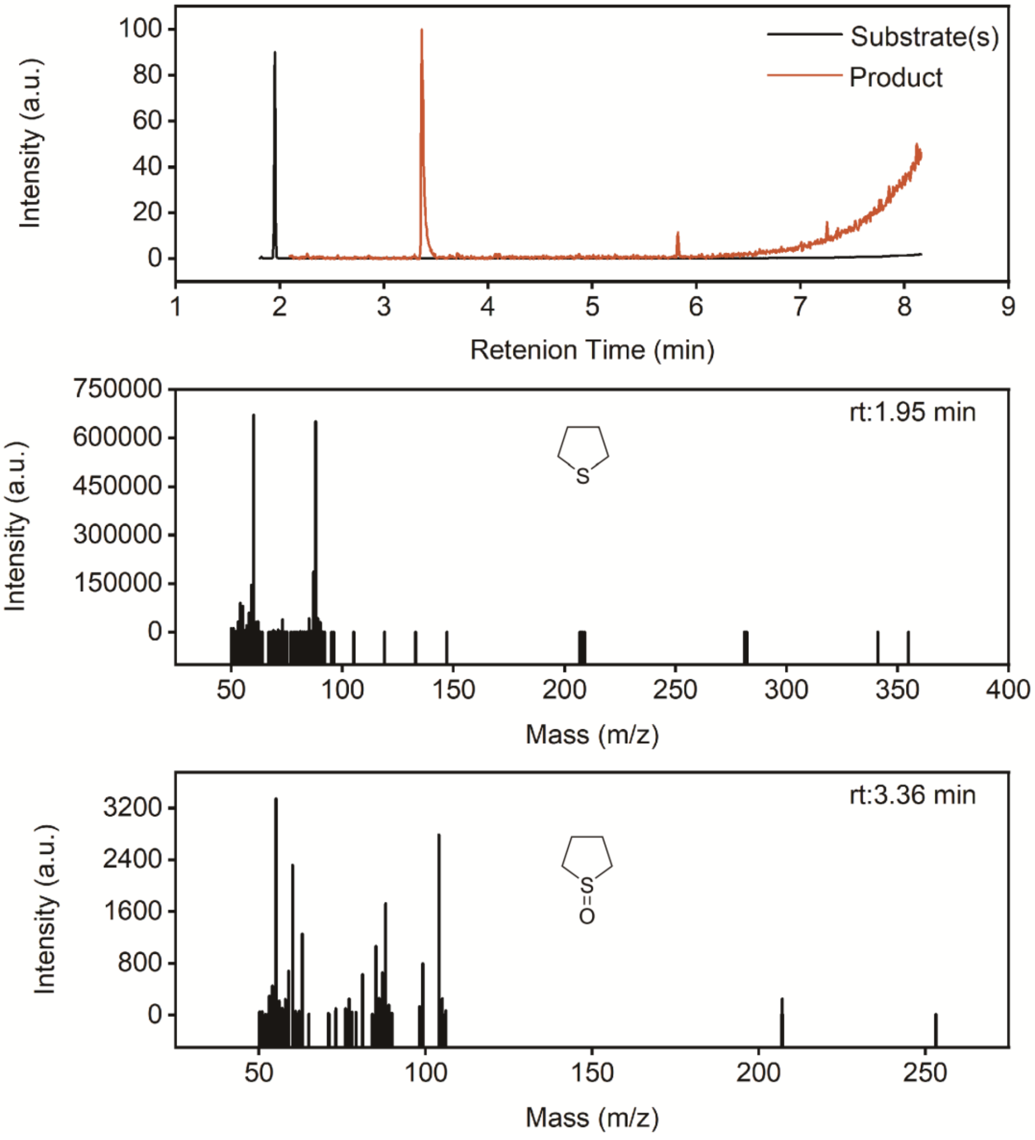

Supplementary Figure 25. GC-MS spectrum of entry 13

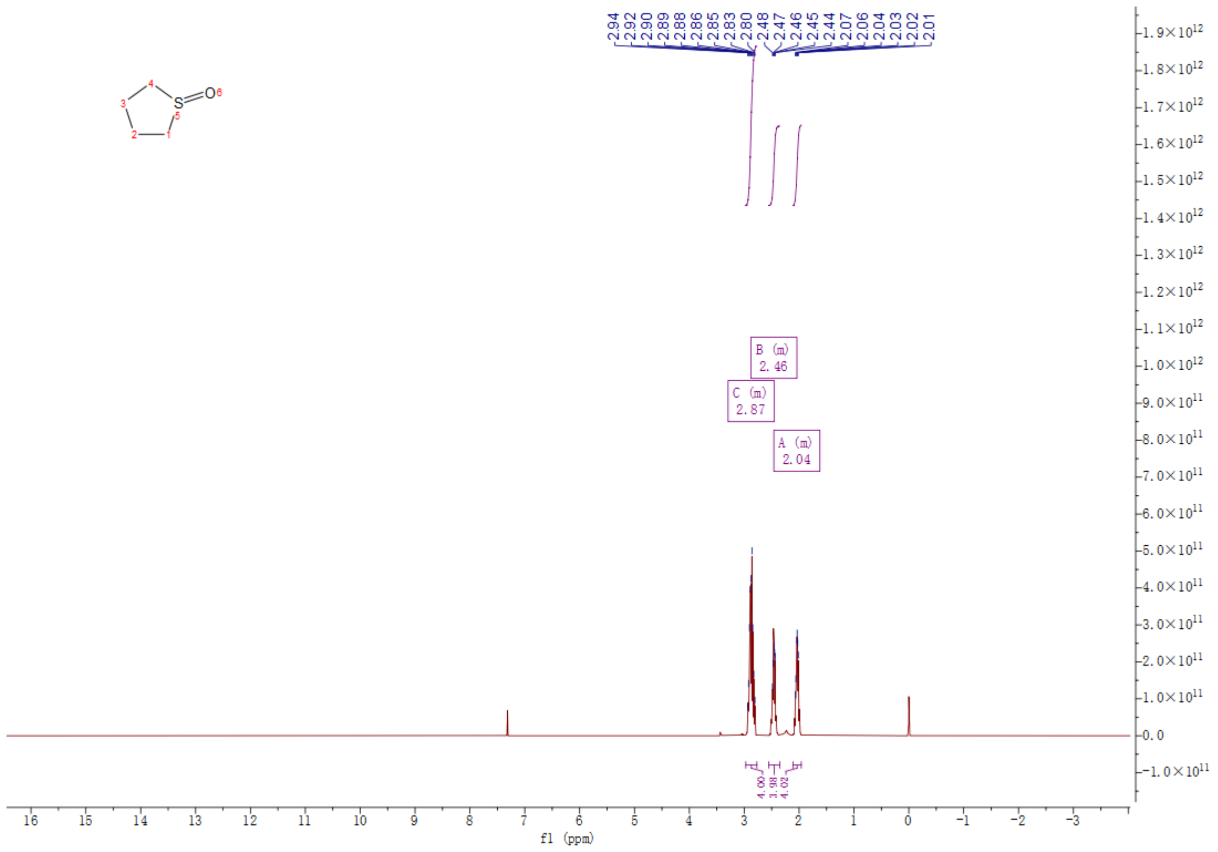

Supplementary Figure 26. NMR spectrum of Tetramethylene sulfoxide

1H NMR (400 MHz, CDCl3) δ 2.97 – 2.77 (m, 4H), 2.55 – 2.35 (m, 4H), 2.11 – 1.96 (m, 4H).

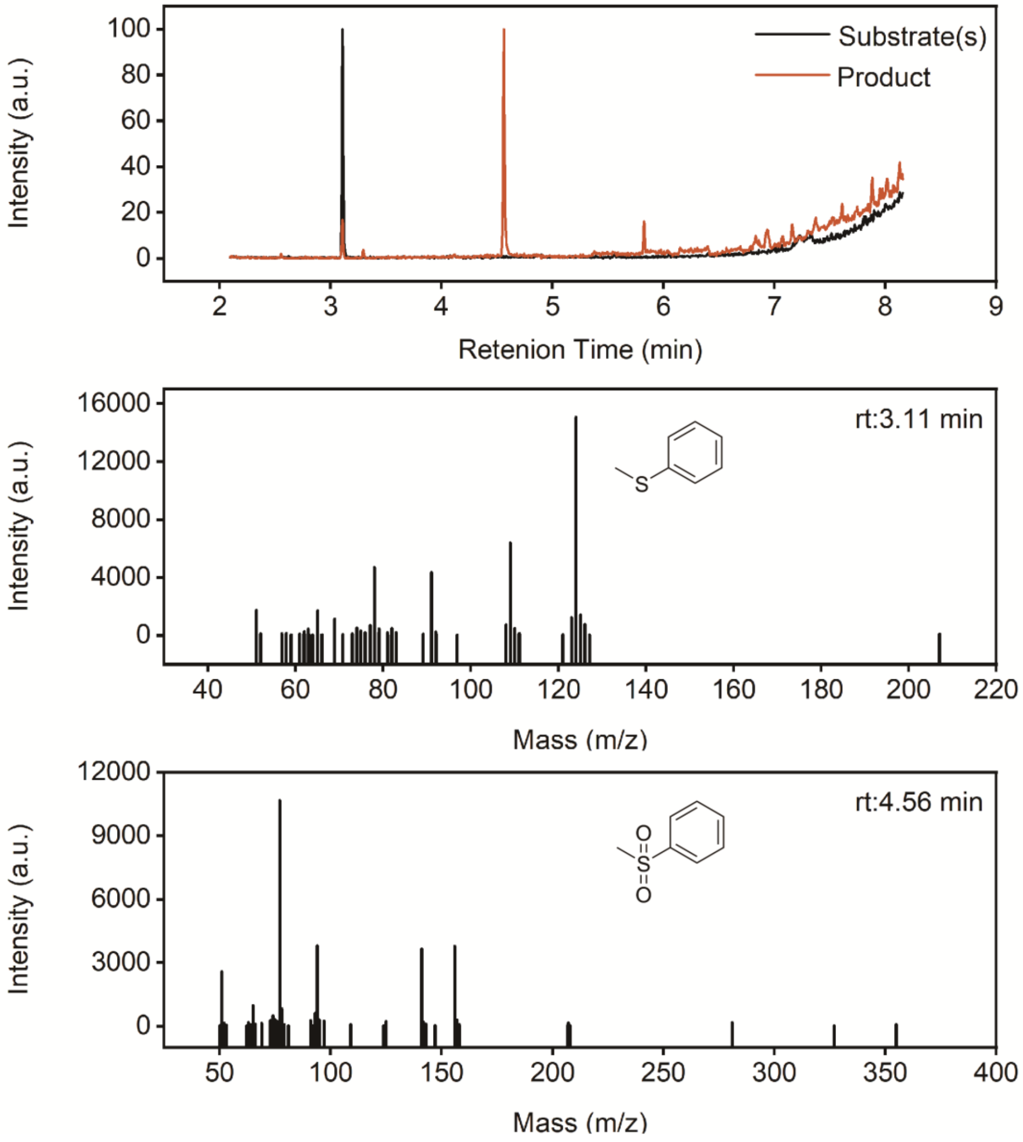

Supplementary Figure 27. GC-MS spectrum of entry 14

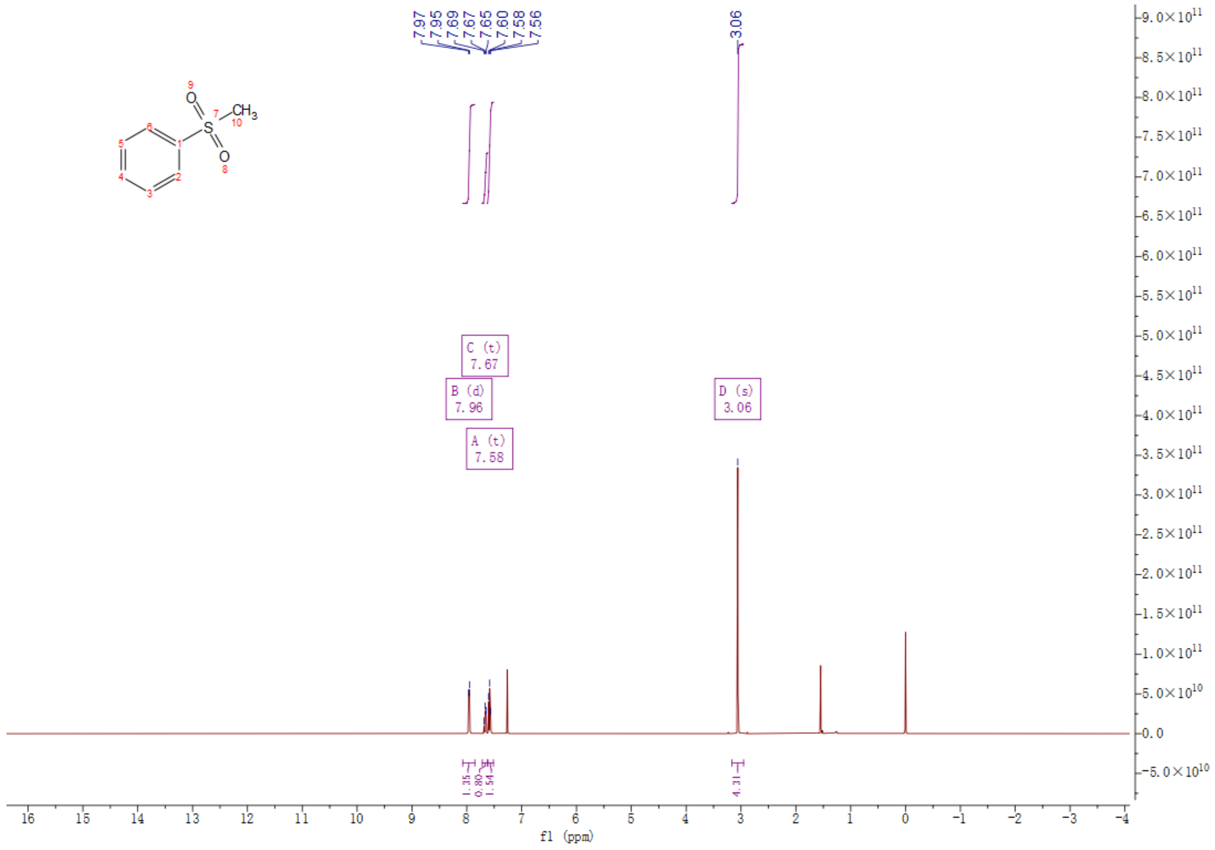

Supplementary Figure 28. NMR spectrum of Methyl phenyl sulfone

1H NMR (400 MHz, CDCl3) δ 7.96 (d, J = 7.0 Hz, 1H), 7.67 (t, J = 7.4 Hz, 1H), 7.58 (t, J = 7.4 Hz, 2H), 3.06 (s, 4H).

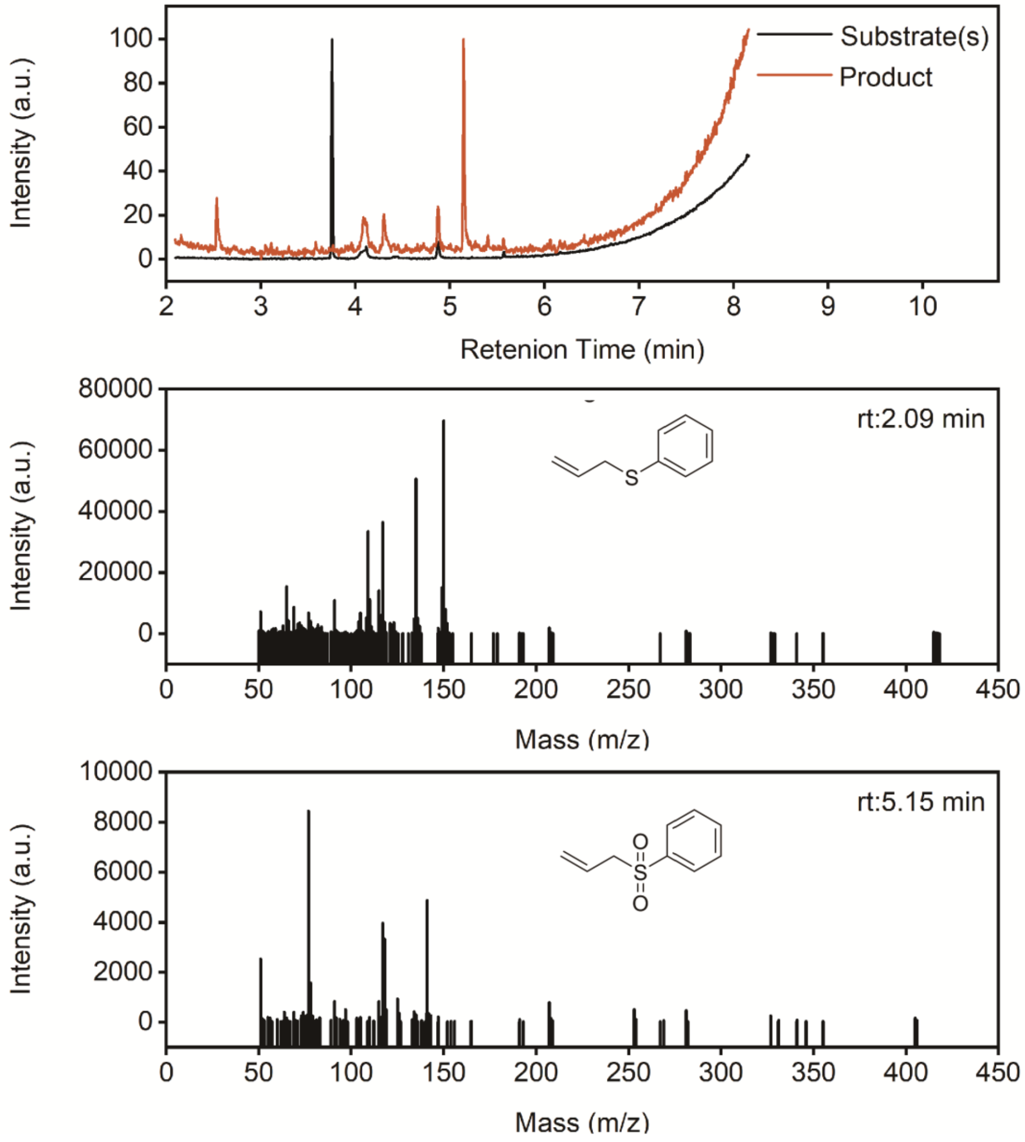

Supplementary Figure 29. GC-MS spectrum of entry 15

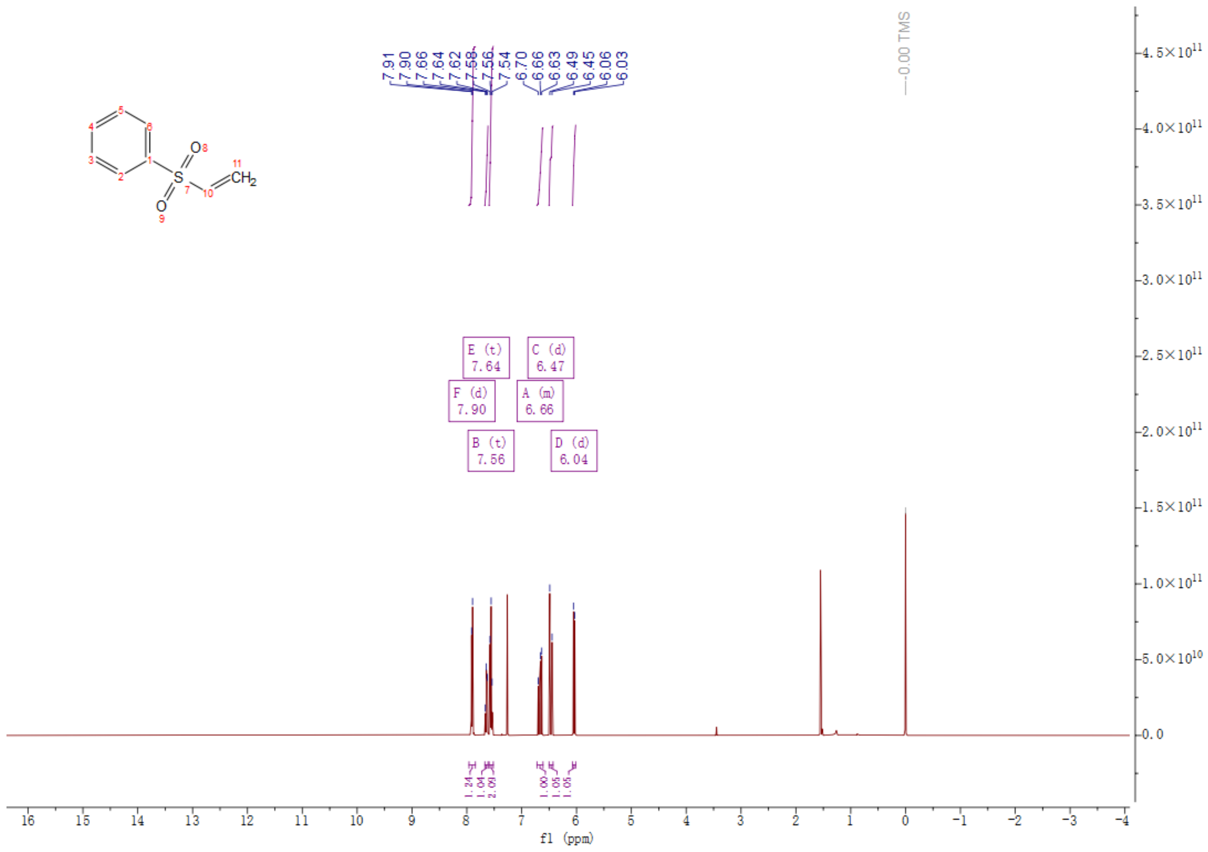

Supplementary Figure 30. NMR spectrum of Phenyl vinyl sulfone

1H NMR (400 MHz, CDCl3) δ 7.90 (d, J = 7.0 Hz, 1H), 7.64 (t, J = 7.4 Hz, 1H), 7.56 (t, J = 7.4 Hz, 2H), 6.72 – 6.61 (m, 1H), 6.47 (d, J = 16.6 Hz, 1H), 6.04 (d, J = 9.8 Hz, 1H).

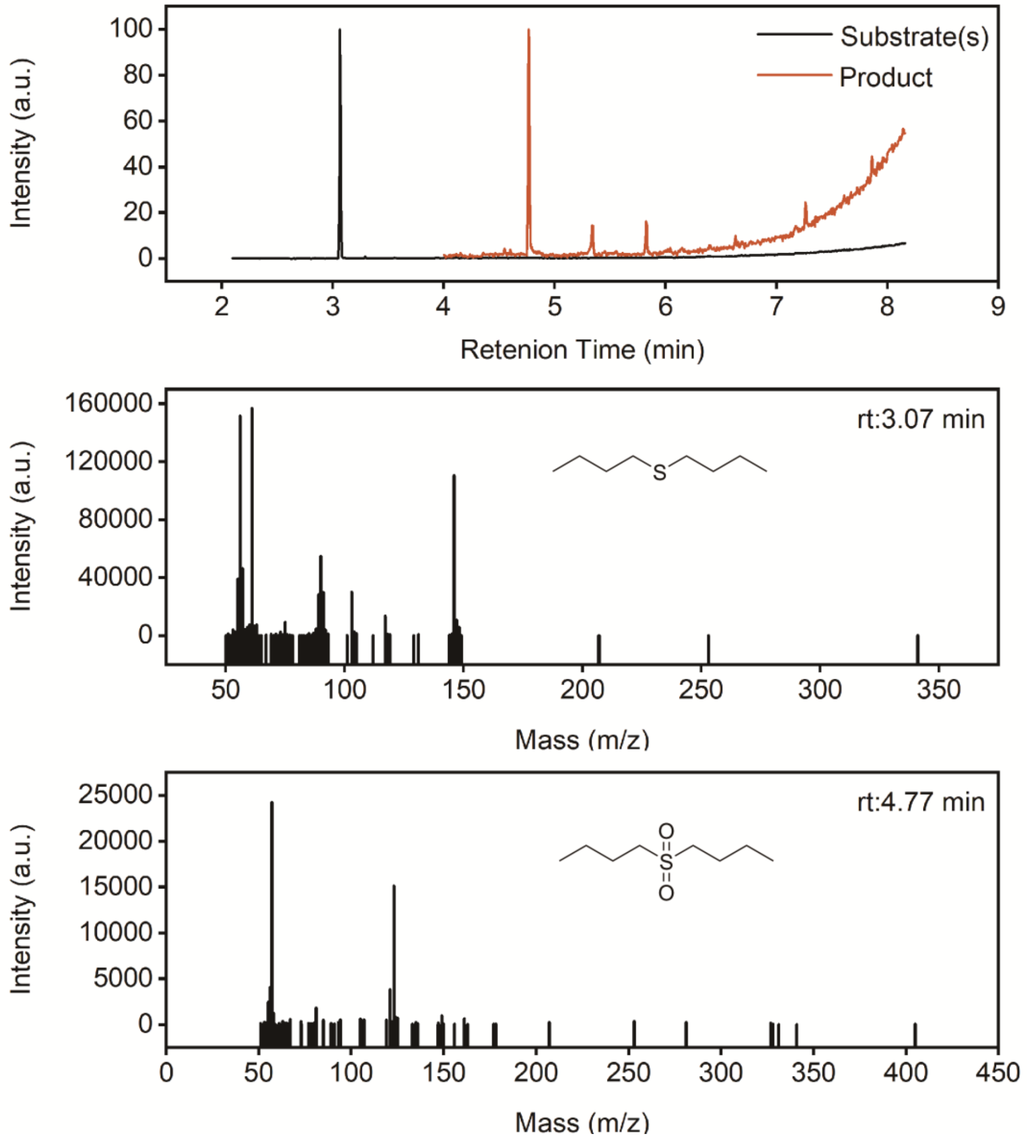

Supplementary Figure 31. GC-MS spectrum of entry 16

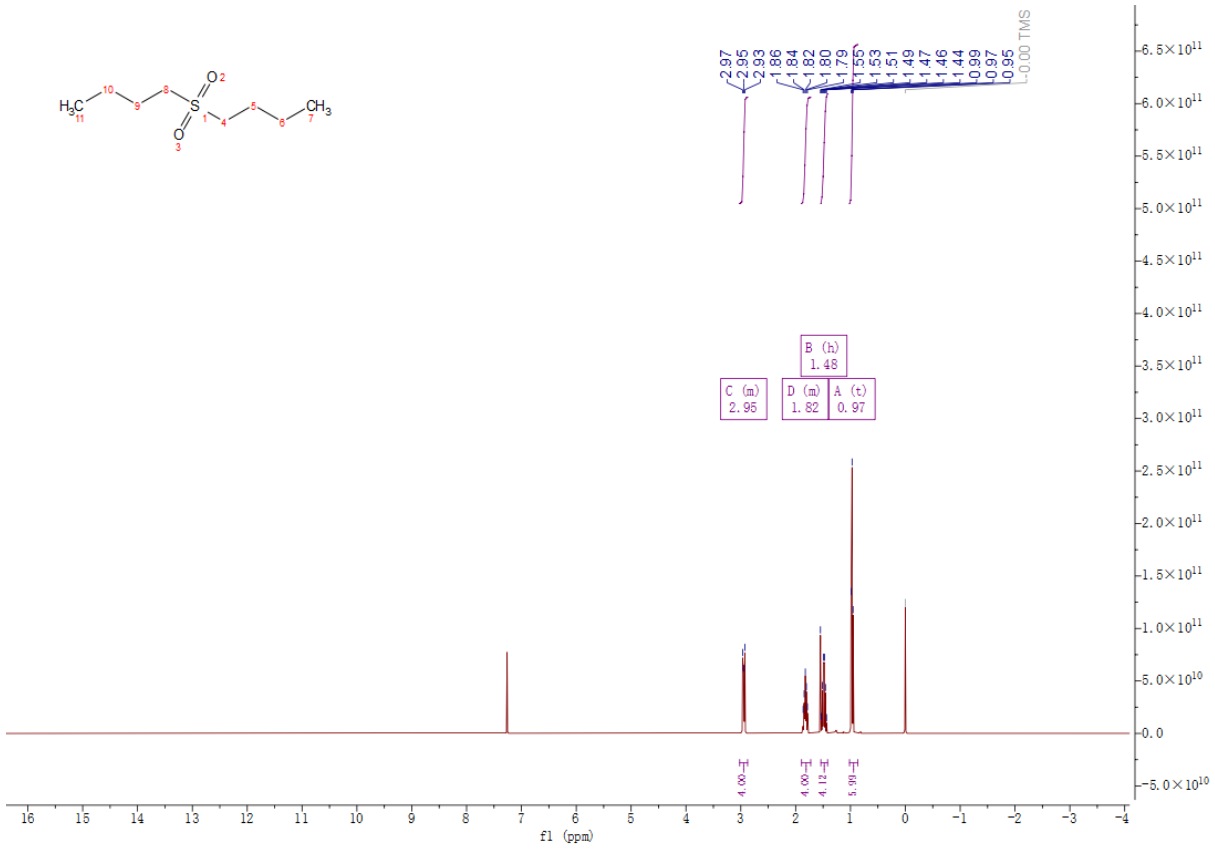

Supplementary Figure 32. NMR spectrum of Di-n-butyl-disulfide

1H NMR (400 MHz, CDCl3) δ 3.02 – 2.88 (m, 4H), 1.89 – 1.73 (m, 4H), 1.48 (h, J = 7.4 Hz, 4H), 0.97 (t, J = 7.3 Hz, 6H).

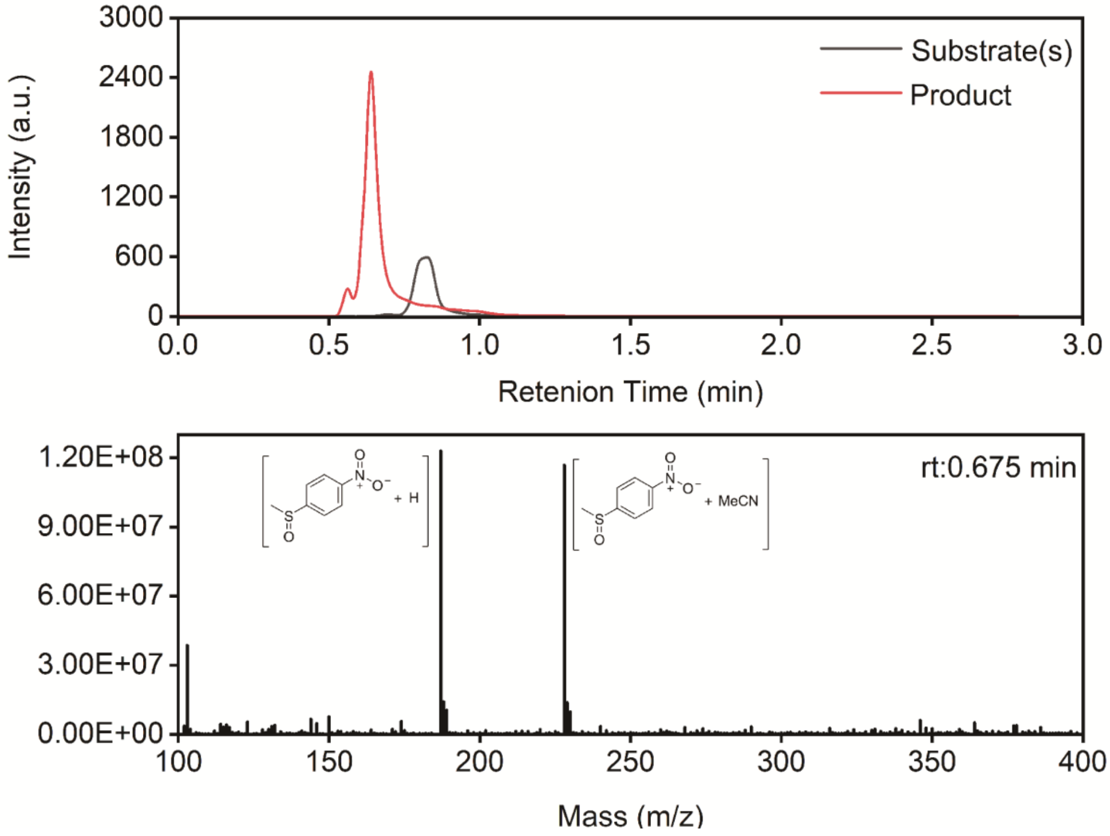

Supplementary Figure 33. UPLC-MS spectrum of entry 17

Cyclicity and stability of cellular reactor

Supplementary Table 8. Cycling performance of the cellular reactor

Cycle	Time 1	Conversion rate 1	Time 2	Conversion rate 2
1	60 min	51.7%	180 min	90.0%
2	60 min	54.7%	180 min	94.3%
3	60 min	57.0%	180 min	94.4%
4	60 min	60.5%	180 min	95.7%
5	60 min	62.6%	180 min	95.2%

A mixture of 12.5 mmol dihydropyran and 12.5 mmol benzyl alcohol (1.0 equiv) was added to 50 mL of cyclohexane. Then, 1 M H_2SO_4 was adsorbed into a 6-leaf cellular reactor and introduced into the organic phase with stirring at 50 rpm. The reaction was conducted in a custom-made three-neck flask heated at 60°C. After each reaction, the cellular reactor was thoroughly washed and dried before proceeding to the next catalytic reaction. The reactor type is Reactor-C in Supplementary Table 4.

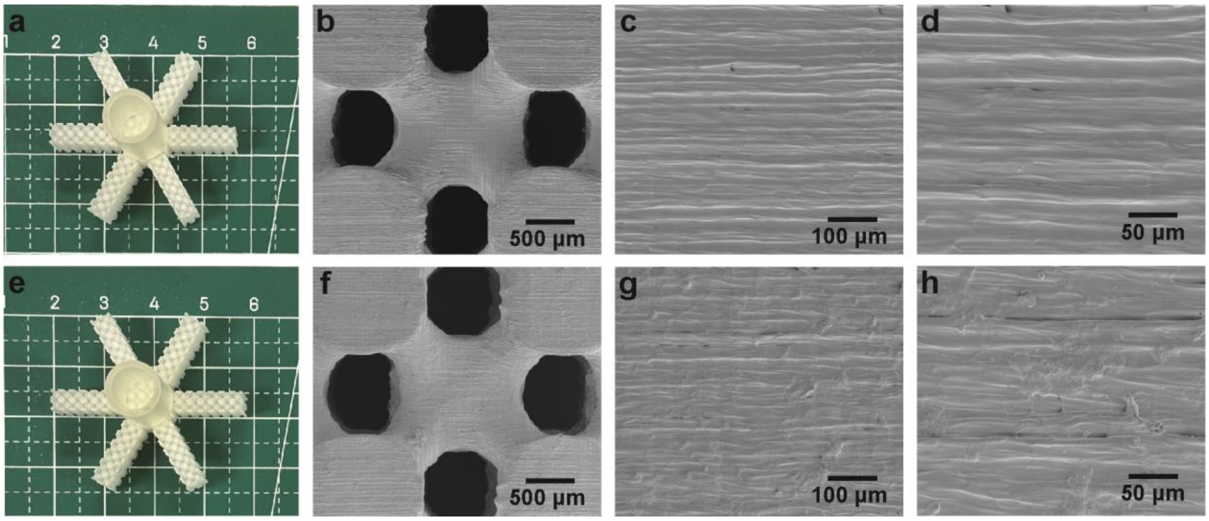

Supplementary Figure 34. Photographs and SEM images of the cellular reactor before and after the cycling reaction (a-d) raw state, not processed in any way (e-h) after cyclic reactions

It is observable that after five cycles, the cellular reactor underwent minimal changes in appearance (Supplementary Figs. 34a and e). The overall structure remained intact (Supplementary Figs. 34b and f), with only slight fine lines appearing on the surface microstructure (Supplementary Figs. 34c, d, g and h). The degree of corrosion observed is entirely acceptable, considering the number of cycles it underwent.

Even after five cycles of use, the cellular reactor remained in excellent condition, preserving its capacity to facilitate liquid-liquid two-phase interface formation. It led to a notable increase in the cellular reactor's utilization efficiency and improved the reaction's economic feasibility.

To further investigate whether different resins are suitable for manufacturing cellular reactors, two other commercial resins from the same company were purchased and used to manufacture cellular reactors in the same manner. Information on the three resins can be found in the table below.

Supplementary Table 9. Introduction to the three resins

Name	Part Number	Description
Clear Resin v4	FLGPCL04	Clear resin, general purpose
Tough 2000 Resin v1	RS-F2-TO20-01	High strength resin
High Temp Resin v2	RS-F2-HTAM-02	High-temperature resistant resin

Photos of cellular reactors manufactured with Clear Resin (CR), Tough 2000 Resin (TR) and High Temp Resin (HR) can be referred to in Supplementary Table 4. Addition reactions between dihydropyran and benzyl alcohol were conducted using the above two resins.

Supplementary Table 10. Catalytic performance of cellular reactors fabricated with three resins

Reactor	Conversion rate (%)			
	30 min	60 min	150 min	300 min
Reactor-C	23.2	53.3	93.5	99.2
Reactor-D	23.7	74.8	98.5	98.7
Reactor-E	51.6	85.8	96.5	98.6

Dihydropyran (12.5 mmol) and benzyl alcohol (12.5 mmol, 1.0 equiv) were combined in 50 mL of cyclohexane. A 1 M H_2SO_4 solution was adsorbed into the cellular reactor and inserted into the organic phase, with stirring at 50 rpm. The reactions were conducted in custom-made three-neck flasks heated to 60°C. Cellular reactors fabricated with three resins were used separately.

Catalytic reactions conducted with high-strength and high-temperature resins achieved results consistent with those from clear resins, and in some cases, the outcomes were even better. The incorporation of two additional resins expanded the material repertoire, confirming that a broader range of materials can be utilized for manufacturing cellular reactors. It further illustrates the versatility of the cellular reactor's application.

To further investigate the corrosive effects of various components on cellular reactor materials, flat cubic samples made from the aforementioned three resins were

3D printed, with dimensions of 10 mm x 10 mm x 2 mm. The flat structure facilitates SEM testing and allows a more accurate comparison of surface morphology changes before and after treatment.

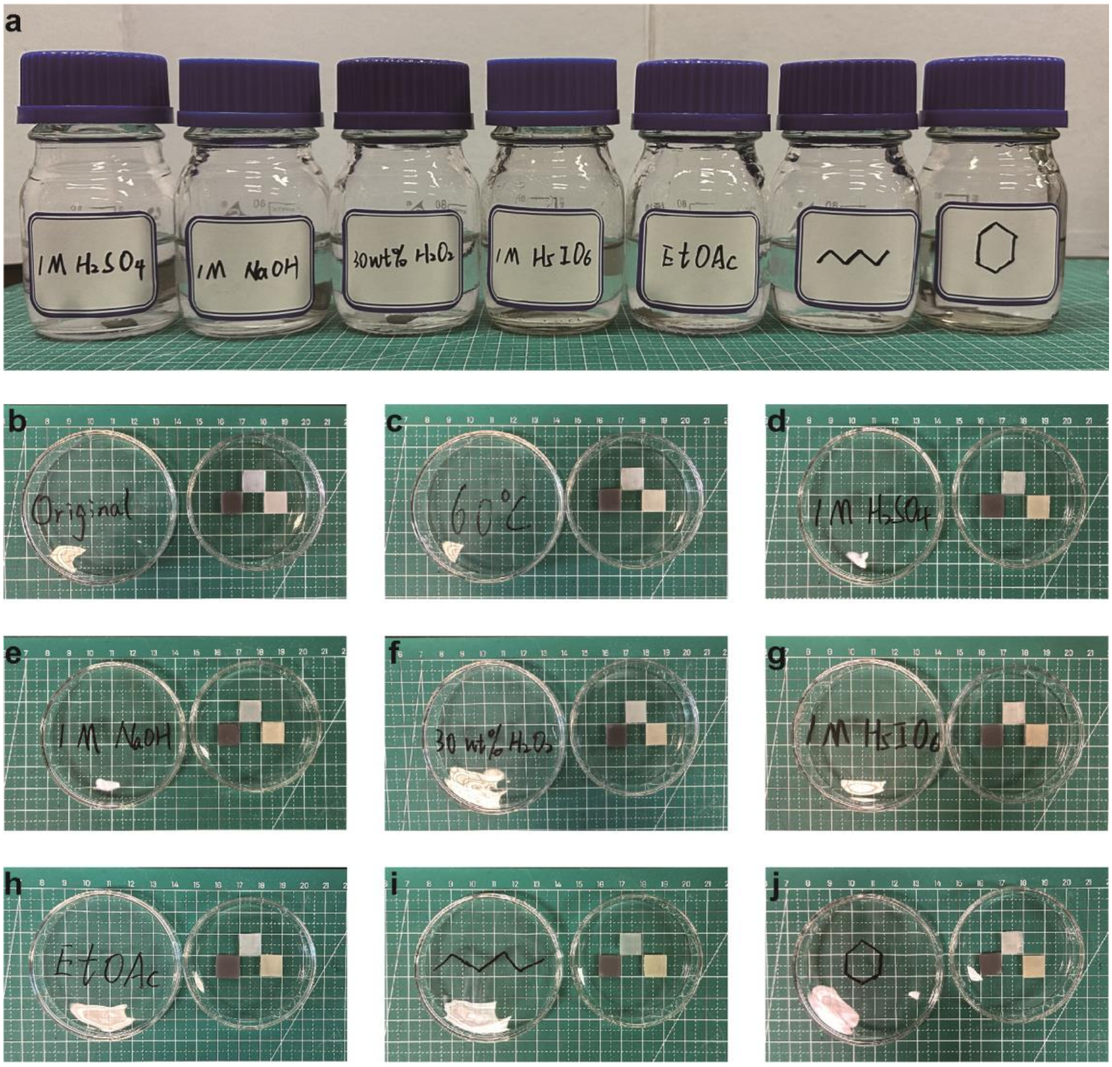

Supplementary Figure 35. Corrosion resistance testing of printed samples from three resins: (a) Small pieces of the three resins were placed in sample bottles containing 50 mL of corrosive liquid and heated at 60°C for 12 hours. (b) Appearance of the small pieces in their original state, untreated. (c) Appearance of the small pieces after heating in a glass surface dish. (d-j) Appearance of the small pieces after treatment with various corrosive liquids.

The following test was conducted to observe the potential corrosion of cellular reactor materials by corrosive components during the catalytic reaction process. Each

sample bottle (100 mL) contained three small pieces printed from different resins and 50 mL of corrosive liquid, with parallel tests conducted using seven different corrosive liquids. Additionally, to assess the impact of heating on the cellular reactor, three small pieces were placed in a glass surface dish and heated alongside the aforementioned seven sample bottles in an oven at 60°C for 12 hours. The seven corrosive liquids tested were 1M H_2SO_4, 1M NaOH, 30 wt% H_2O_2, 1M H_5IO_6, ethyl acetate, n-hexane, and cyclohexane.

Initially, from a visual perspective, whether heated directly or in corrosive liquids, there were no significant changes in the appearance of the small pieces before and after treatment (Supplementary Figs. 35 b-j).

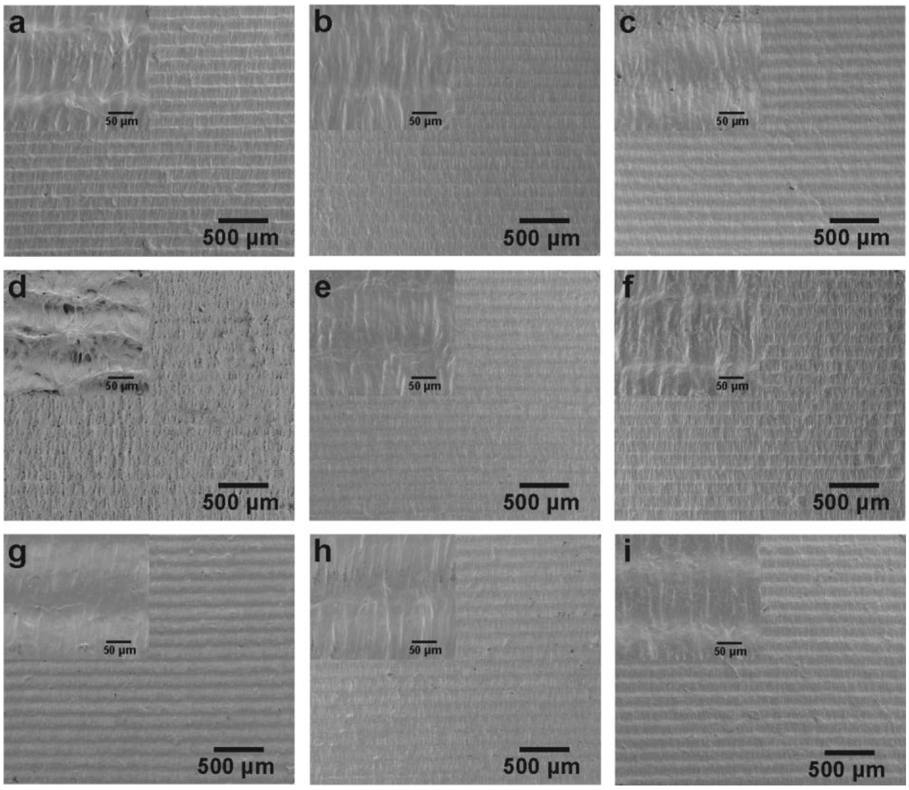

Supplementary Figure 36. SEM images of clear resin printed sheets (a) (a) raw state,

without any processing (b) heated in glass surface dish (c) processed by 1M H_2SO_4 (d) processed by 1M NaOH (e) processed by 30 wt% H_2O_2 (f) processed by 1M H_5IO_6 (g) processed by ethyl acetate (h) processed by n-hexane (i) processed by cyclohexane.

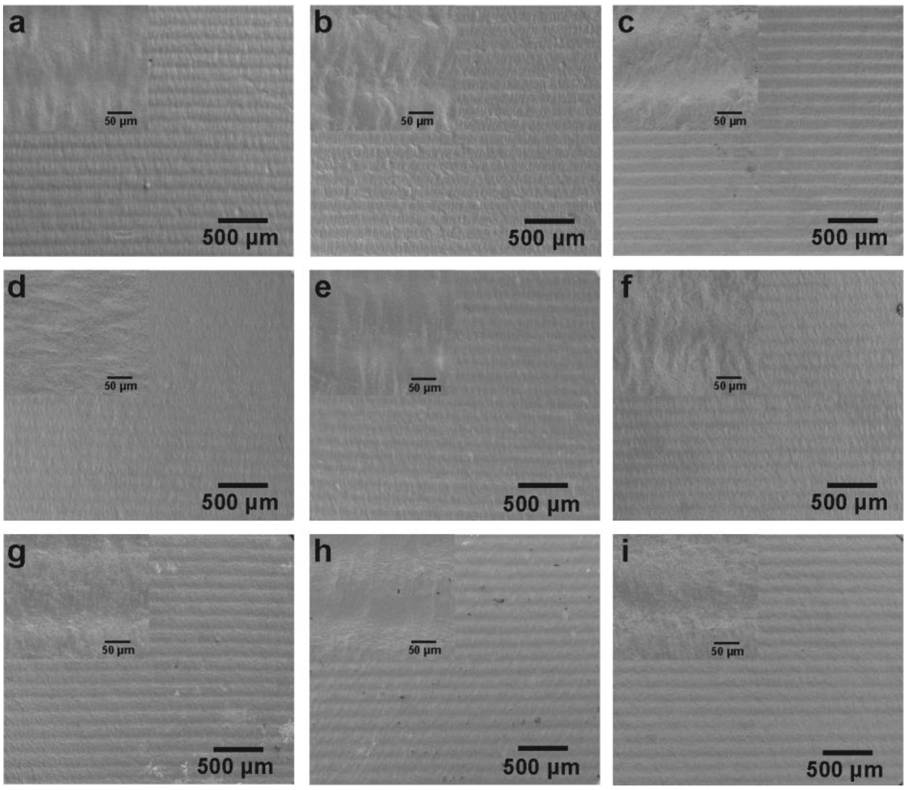

Supplementary Figure 37. SEM images of tough-2000 resin printed sheets (a) raw state, without any processing (b) heated in glass surface dish (c) processed by 1M H_2SO_4 (d) processed by 1M NaOH (e) processed by 30 wt% H_2O_2 (f) processed by 1M H_5IO_6 (g) processed by ethyl acetate (h) processed by n-hexane (i) processed by cyclohexane.

From the SEM images, the small pieces before treatment (Supplementary Fig.

36a) exhibited a grooved structure on the surface due to the layer-by-layer manufacturing process in additive manufacturing. After treatment, the small clear resin pieces (Supplementary Figs. 36b-i), compared to the untreated ones, maintained their grooved structure in the vast majority, indicating that the aforementioned treatments had minimal corrosive effects on the clear resin. The only exception was the small piece treated with NaOH solution, which showed a more flattened grooved structure, suggesting that clear resin has a lower tolerance to strong alkalis.

The situation of the high-strength resin pieces was similar to that of the clear resin pieces (Supplementary Figs. 37a-i), with strong alkalis causing some degree of corrosion to the high-strength resin.

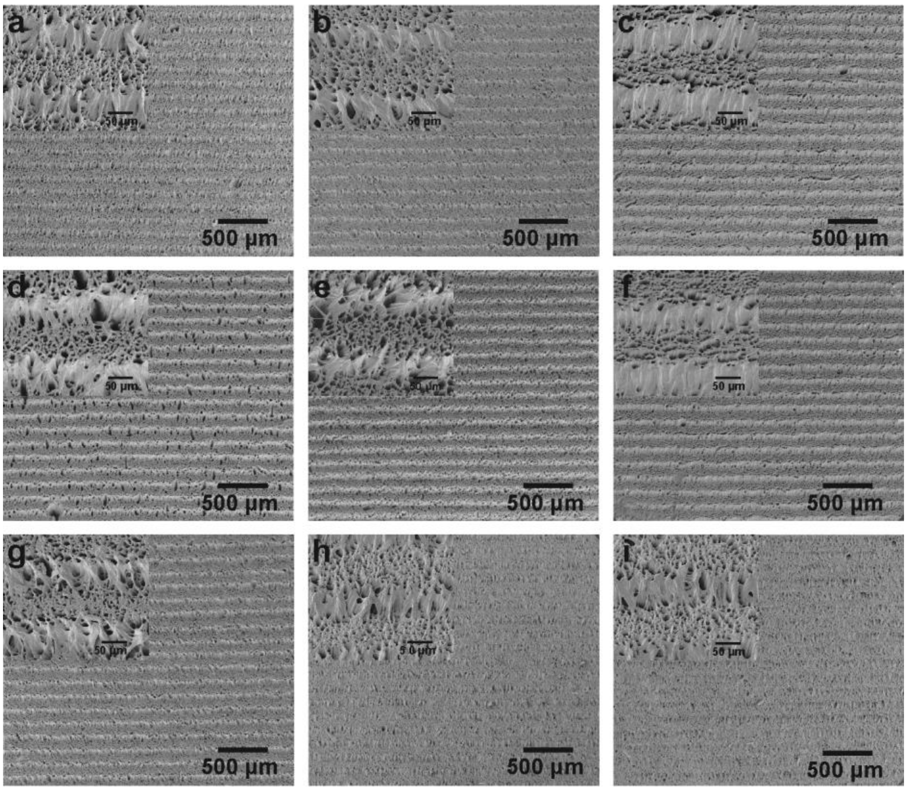

Supplementary Figure 38. SEM images of high-temp resin printed sheets (a) raw state,

without any processing (b) heated in glass surface dish (c) processed by 1M H_2SO_4 (d) processed by 1M NaOH (e) processed by 30 wt% H_2O_2 (f) processed by 1M H_5IO_6 (g) processed by ethyl acetate (h) processed by n-hexane (i) processed by cyclohexane.

Surprisingly, for the high-temperature resistant resin, the surfaces of the treated small pieces (Supplementary Figs. 38 b-i) showed almost no changes compared to the untreated ones (Supplementary Fig. 38a). Only the pieces treated with n-hexane and cyclohexane (Supplementary Figs. 38 h-i) slightly collapsed in the surface grooves, but the microstructure (shown in the small image in the top left corner) remained unchanged. These observations indicate that the high-temperature resistant resin can withstand corrosion from all the aforementioned solutions, making it a widely applicable resin material.

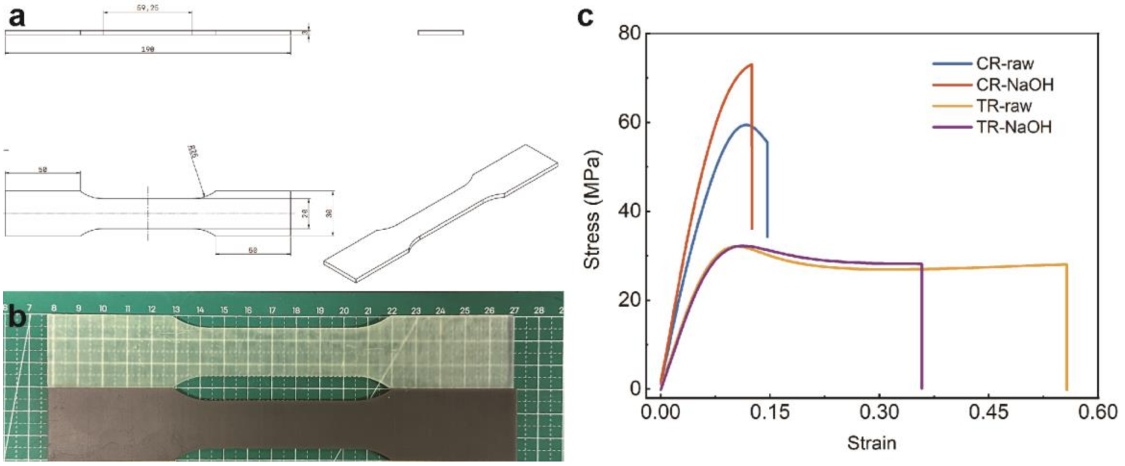

Supplementary Figure 39. presents the testing of resin materials for NaOH tolerance: (a) the blueprint of the standard test specimen, (b) a photograph of the standard test

specimen, and (c) the strain-stress curves of the resin materials before and after NaOH treatment.

Prefix CR represents Clear Resin v4, and TR stands for Tough 2000 Resin v1. The suffix raw indicates the original, untreated standard test specimens, while NaOH denotes the specimens after being heated in 1M NaOH at 60°C for 12 hours. In the strain-stress curves, stress increases with strain during the initial stretching phase, characterizing the elastic deformation process. When strain reaches a certain critical value, stress decreases with increasing strain, marking the plastic deformation stage, where the deformation becomes irreversible. The stress drops precipitously upon reaching another critical strain value, indicating the specimen's breakage. For the standard specimens of Clear Resin, both elastic and plastic deformation capabilities decreased after NaOH treatment, yet they retained considerable elastic deformation capacity. Similarly, the standard specimens of Tough Resin yielded consistent conclusions.

Supplementary Table 11. Comparison of the solid cellular reactor with two types of commercially available reactors

Time (min)	Conversion rate (%)			
	Reactor-C	Reactor-F	Reactor-G	Reactor-H
30	37.6	1.9	4.1	3.8
60	64.8	2.5	6.9	8.1
90	84.2	4.9	8.6	10.2

Dihydropyran (12.5 mmol) and benzyl alcohol (12.5 mmol, 1.0 equiv) were combined in 50 mL of cyclohexane. An equivalent volume of 1 M H_2SO_4 solution, matching the liquid holding capacity of the cellular reactor, was added into the cellular reactor and inserted into the organic phase, stirring at 50 rpm. The reactions were conducted in custom-made three-neck flasks heated to 60°C.

It is evident that, whether employing a solid stirring paddle without cell units or a commercially available generic stirring paddle, the reaction rate is significantly slower than when using the cellular reactor. Experimental observations indicate that the design and use of the cellular reactor are necessary.

Particle Image Velocimetry

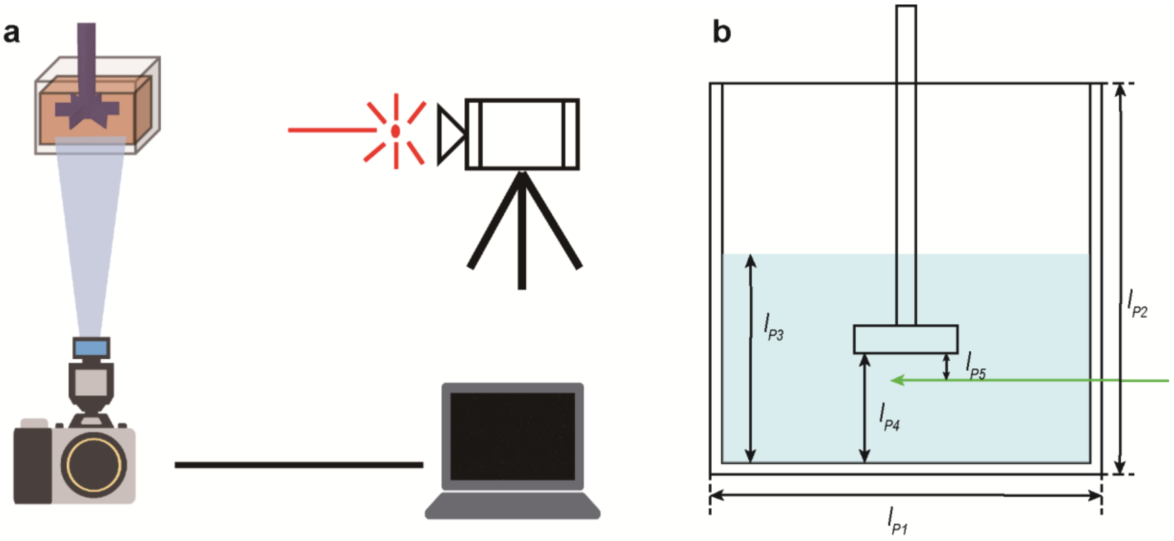

Supplementary Figure 40. Schematic of Particle Image Velocimetry (a) Diagram of the setup (b) Frontal view with dimension parameters annotated. The particle image velocimetry setup consists of a laser, a high-speed camera, and a synchronizer, including a stirring device related to the cellular reactor. The involved dimensions are as follows:

Supplementary Table 12. Annotation of dimensions and comments.

Symbol	Length	Description
l_{P1}	12 cm	Length of the container
l_{P2}	12 cm	Width of the container
l_{P3}	7 cm	Height of liquid
l_{P4}	4 cm	Distance between the lower edge of the cellular reactor and the bottom of the liquid
l_{P5}	1 cm	Distance between the lower edge of the cellular reactor and the incident laser light

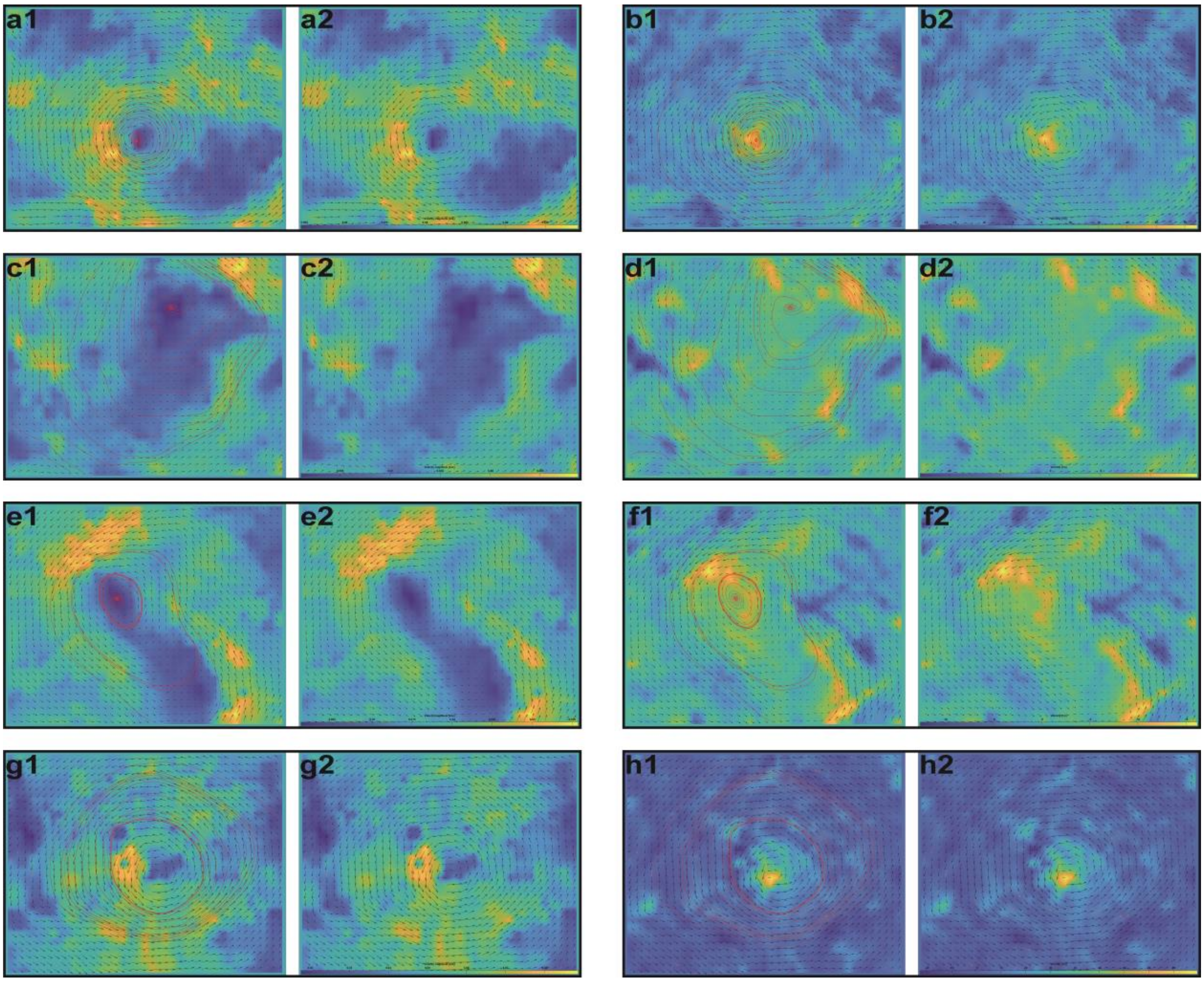

Supplementary Figure 41. Particle Image Velocimetry data for the cellular reactor (Reactor-C) at 50 rpm includes (a) velocity map and (b) vorticity map. For the commercially available generic stirring paddle 1 (Reactor-G) at 50 rpm: (c) velocity map and (d) vorticity map. For the commercially available generic stirring paddle 2 (Reactor-H) at 50 rpm: (e) velocity map and (f) vorticity map. For the cellular reactor (Reactor-C) at 100 rpm: (g) velocity map and (h) vorticity map.

The classical PIV (Particle Image Velocimetry) test procedure is as follows: First, assemble the apparatus, ensuring the relative positions of each device are fixed, using

a level to maintain the laser's perpendicular orientation to the incident plane and the high-speed camera. Fluorescently labeled tracer particles are added to hexane and sonicated to ensure uniform dispersion. The aforementioned mixture is then added to a container, followed by water adsorption into the cellular reactor, which is then inserted into the corresponding position in the container and rotated at an appropriate angular velocity. After stabilizing the rotation for a period, the laser and camera are activated to collect images. The collected images are analyzed using the free, open-source PIV Lab code in Matlab software to determine fluid motion based on the flow of the particles, obtaining images and data on velocity and vorticity. The procedure for using a generic stirring paddle is identical to the above, except that the cellular reactor is replaced with a generic stirring paddle, and water is not adsorbed.

Supplementary Table 13. Maximum velocity and maximum vorticity as measured by PIV

Reactor	No.	Rotation speed	Maximum velocity (m/s)	Maximum vorticity (1/s)
Reactor-C	a&b	50 rpm	0.04	32
Reactor-G	c&d	50 rpm	0.03	15
Reactor-H	e&f	50 rpm	0.036	16
Reactor-C	g&h	100 rpm	0.09	80

Based on the data in Supplementary Table 6, at the same rotational speed, the cellular reactor can induce greater velocity and larger flow field disturbances in the reaction system, outperforming the fluid dynamics capabilities of two types of generic stirring paddles. Furthermore, the cellular reactor possesses liquid retention and separation functions that generic stirring paddles lack. Therefore, the design of the cellular reactor is meaningful.

Effect of rotation speed on reaction rate

The cellular reactor was utilized for chemical reactions under various stirring conditions, exemplified by the acid-catalyzed addition reaction of dihydropyran to alcohol. These conditions included static (0 rpm) and at 50, 100, 150, and 200 rpm.

Supplementary Table 14. Conversion of dihydropyrans at different rotational speeds

Time (min)	Conversion rate (%)				
	0 rpm	50 rpm	100 rpm	150 rpm	200 rpm
30	0.0	6.4	5.1	8.3	8.0
45	1.6	9.2	5.3	8.2	10.0
60	0.7	16.4	12.4	17.5	31.2
90	6.2	20.3	23.1	24.5	49.5
120	26.9	36.8	33.5	33.6	60.9
150	12.1	42.7	45.5	41.2	70.8

Dihydropyran (12.5 mmol) and benzyl alcohol (12.5 mmol, 1.0 equiv) were combined in 50 mL of cyclohexane. A 1 M H_2SO_4 solution was adsorbed into the cellular reactor and inserted into the organic phase, with stirring at 50 rpm. The reactions were conducted in custom-made three-neck flasks heated to 60°C. There are two reactions without and with 0.01 wt% hollow glass bubbles. All reactor types are Reactor-C in Supplementary Table 4.

The reaction rate is very slow in the absence of stirring. At a stirring speed of 200

rpm, liquid leakage occurs within the cellular reactor. It is due to insufficient adhesive forces to counterbalance the increased centrifugal force at high stirring speeds. The reaction rates are relatively similar at lower stirring speeds (50, 100, and 150 rpm). The impact of stirring speed on reaction rate is quite limited at these speeds. This is because increasing the stirring speed primarily enhances the mass transfer rate of substances in the external organic phase. At the same time, the rate-determining step of the interface reaction involves the diffusion of substrates and catalyst components to the interface, not just the mass transfer within the external oil phase. Therefore, increasing the stirring speed does not significantly enhance the reaction rate.

Although the capability of cellular reactors to enhance the rate of chemical reactions under working conditions is limited, their functionality in maintaining interface stability, limiting corrosive components, and greatly reducing separation difficulty is irreplaceable. Therefore, the introduction of cellular reactors serves a meaningful purpose.

Simulation experiments with solid additives

Supplementary Table 15. Simulation experiments with solid additives

Additives	Conversion rate (%)			
	30 min	60 min	120 min	180 min
-	37.6	64.3	91.3	97.5
glass bubbles	38.8	63.3	90.6	97.4

Dihydropyran (12.5 mmol) and benzyl alcohol (12.5 mmol, 1.0 equiv) were combined in 50 mL of cyclohexane. A 1 M H_2SO_4 solution was adsorbed into the cellular reactor and inserted into the organic phase, with stirring at 50 rpm. The reactions were conducted in custom-made three-neck flasks heated to 60 °C. There are two reactions without and with 0.01 wt% hollow glass bubbles. All reactor types are Reactor-C in Supplementary Table 4.

Hollow glass microspheres were purchased from 3M Company, with sizes ranging from 9-25 μm and a density of 0.6 g/cc. They were added at a concentration of 0.01 wt% to 50 mL to simulate solid impurities generated in certain reactions. The reaction rate of reactions with added hollow glass microspheres was very close to those without the microspheres. Therefore, the cellular reactor demonstrates good applicability for reactions that generate precipitate as a by-product.

Cross-contamination between water phase and organic phase

The solubility differences between the two phases are extremely large for completely immiscible two-phase systems, such as water with n-hexane or cyclohexane. The solubility of n-hexane in water is only 0.014%, so cross-contamination between the two phases can be considered negligible. For partially miscible systems, like water and EtOAc, the relative contents of each phase were measured. The amount of EtOAc in water was determined by Total Organic Carbon (TOC) analysis, while the water content in EtOAc was measured using Karl Fischer method.

The cellular reactor first adsorbs water and is then inserted into 50 mL of ethyl acetate after stirring at 50 rpm at room temperature. For 5 hours, the water phase is separated from the cellular reactor, yielding sample S16-1W, with the organic phase being sample S16-1EA. Using a commercially available stirrer, an amount of water equal to the holding capacity of the cellular reactor is added to 50 mL of ethyl acetate. After stirring at 50 rpm at room temperature for 5 hours, the water phase is extracted to obtain sample S16-2W, with the organic phase being sample S16-2EA. The process for obtaining sample 3 is identical to that for sample 2, except the stirring speed is changed from 50 to 1000 rpm. After stirring and allowing the phases to separate, the water phase obtained is sample S16-3W, with the organic phase sample S16-3EA. The cellular reactor is made of resin material and could also be a potential organic carbon source. Therefore, we placed the cellular reactor in 50mL of water and stirred

at 50 rpm at 60℃ for 5 hours to verify whether the cellular reactor would contaminate the water phase, with the sample being labeled as S16-4W.

For measuring the ethyl acetate content in water, a Total Organic Carbon (TOC) analyzer was used to approximate the ethyl acetate content by measuring the total organic carbon present. The TOC analyzer employs a combustion method, oxidizing all organic matter to carbon dioxide, determining the organic carbon content.

Supplementary Table 16. Total organic carbon in water

Sample	DI Water	S16-1W	S16-2W	S16-3W	S16-4W
TOC (ppm)	2.13	1467	2425.5	1366	4.57

The total organic carbon (TOC) content in the pure deionized water used throughout the experiment was measured to be 2.13 ppm as a blank control. Sample 4W showed a TOC content of 4.57 ppm for a second blank control, indicating that the cellular reactor released a minimal amount of organic substances into the water. It further demonstrates the stability of the cellular reactor in water. Under ambient temperature and stirring at 50 rpm, sample 1, obtained using the cellular reactor, had a lower ethyl acetate content in water compared to sample 2, obtained using a commercial stirrer, signifying that the use of the cellular reactor can significantly reduce cross-contamination between the two phases.

The Karl Fischer method was employed to measure the water content in ethyl

acetate. Given that the water content in the sample is not trace but rather minute, volumetric analysis is deemed suitable for this system. A two-component Karl Fischer reagent (comprising solvent and titrant) was utilized, with pre-titration of the solvent conducted prior to experimentation to establish a blank control for moisture content. A specific volume of the sample was introduced into the solvent, followed by incremental titrant addition with stirring to ensure complete reaction. Upon color change indicating the endpoint of titration, the volume difference of titrant before and after titration (ΔV) was recorded and used to calculate the water content. Since commercially available ethyl acetate may also contain water, immediate water content testing was performed upon extraction from the reagent bottle as a control group.

The water content is calculated using the following formula.

$$C_W = \frac{\Delta V \times Tit}{V_{EA}}$$

In this equation, ΔV represents the volume difference of the titrant before and after titration, V_{EA} denotes the volume of the sample added (1 mL), Tit signifies the titer (5000 ppm, expressed as H_2O), and C_W denotes the water content of the sample.

Supplementary Table 17. Water content of ethyl acetate determined by Karl Fischer volumetric titration

Sample	EtOAc	S16-1EA	S16-2 EA	S16-3 EA
$\boldsymbol{\Delta V}$ (mL)	0.08	0.17	0.24	0.35
$\boldsymbol{C_W}$(ppm)	400	850	1200	1750

At room temperature, the utilization of cellular reactors resulted in an increase in

water content, from 400 ppm to 850 ppm, which remained below that observed with commercially available stirrers (1200 ppm) and notably lower than in cases of vigorous agitation (1750 ppm). It indicates that while the cellular reactor increases the interfacial area, it does not introduce additional cross-contamination between the two phases. On the contrary, compared to vigorous stirring, the cellular reactor reduces cross-contamination between the phases and facilitates rapid separation afterward, making it an advanced tool for chemical processes.

Additionally, after the reaction, apart from organic solvents, the water phase inside the cellular reactor may also contain a small amount of substrates/products that are slightly soluble in water. We conducted thermogravimetric analysis on the post-reaction aqueous phase to represent the degree of cross-contamination in the water phase accurately. Samples of the water phase from the acid-catalyzed addition of dihydropyran to alcohol and the base-catalyzed Knoevenagel condensation reaction were tested. The testing was conducted in a nitrogen atmosphere, starting from room temperature and heating at a rate of 3 °C /min to 400°C.

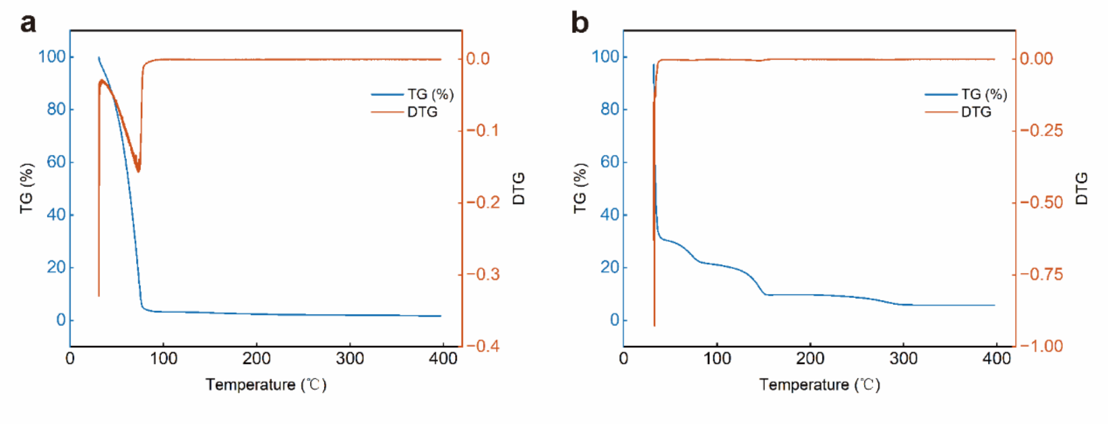

Supplementary Figure 42. Thermogravimetric curve of the aqueous phase after reaction

In the water sample test following acid catalysis (Supplementary Fig. 42a), the mass loss at 75.5°C is attributed to water, leaving 9.4% of the initial mass. Although this temperature does not reach the boiling point of water, the slow rate of temperature increase and the continuous flow of N_2 over the sample accelerated the loss of water. When the temperature reached 100°C, the remaining mass was 3.3% of the initial

mass, indicating that all the water had been lost. At 337°C, the remaining mass was 1.9% of the initial mass, which should correspond to sulfuric acid. Therefore, the mass lost between 100-337°C originated from organic phase components, including substrates and products. It suggests that the degree of cross-contamination between the two phases is very low.

Supplementary Table 18: Boiling points of all substances in the aforementioned system

Compound	CAS No.	Boiling point
Cyclohexane	110-82-7	80.7°C
3,4-Dihydro-2H-pyran	110-87-2	86°C
Benzyl alcohol	100-51-6	205°C
(Tetrahydro-2H-pyran-2-yl)benzyl ether	1927-62-4	~280°C
Sulfuric acid	7664-93-9	337°C
Ethyl acetate	141-78-6	77°C
4-Chlorobenzaldehyde	104-88-1	214°C
Malononitrile	109-77-3	220°C
2-(4-Chlorobenzylidene)propanedinitrile	1867-38-5	~330°C
Potassium carbonate	584-08-7	1689°C (decomposes)

In the water sample test following base catalysis (Supplementary Fig. 42b), three points of slope change were observed. At the first change in slope, the temperature reached 44°C, with the remaining mass being 30.6% of the initial mass, indicating the beginning of the loss of ethyl acetate and water; at the second slope change, the

temperature reached 80°C, with the remaining mass at 22.6% of the initial mass, marking the complete loss of ethyl acetate; at the third change in slope, at 154°C, the remaining mass was 9.7% of the initial mass, indicating the complete loss of water. When the temperature rose to 397°C, the remaining mass was 5.73% of the initial mass. It suggests that the substrates, products, and any potential by-products dissolved in water account for less than 4% of the total mass of the aqueous phase.

Therefore, the degree of cross-contamination between the two phases was determined through a combined analysis using various testing methods including Total Organic Carbon (TOC) analysis, Karl Fischer volumetric titration., and TGA analysis. For partially miscible systems (water and ethyl acetate), the degree of cross-contamination between the phases was similar, whether using the cellular reactor or not. However, the cellular reactor's ability to restrict fluid movement and simplify the subsequent separation of water and oil phases is an advantage that conventional stirring systems cannot offer. Hence, we believe incorporating the cellular reactor into biphasic liquid-liquid reactions is meaningful.

Comparison between the use and non-use of cellular reactor

Calculation of liquid-liquid interfacial area created by cellular reactors with and without cellular units.

The data regarding the interphase interface is derived from calculations. Initially, the system utilized a solid stirring paddle at a low speed (50 rpm). To compare the contact area and characterize the benefits of using the cellular reactor, the volume of the aqueous phase catalytic liquid added was consistent with the liquid holding capacity of the cellular reactor. Within the reaction liquid, the aqueous phase components assume a shape very close to spherical contraction. Hence, we employed the formula for calculating the surface area of a sphere to estimate the contact area between the two phases.

$$S = 4\pi R^2$$

$$V = 4\pi R^2$$

Herein, S represents the surface area of the spherical droplet, R denotes the radius of the spherical droplet, and V signifies the volume of the spherical droplet. It gives an interfacial area of 0.00081 mm^2.

Furthermore, regarding the liquid-liquid catalytic process involving the cellular reactor. In practice, the internal phase liquid is adsorbed into and fills the interior of the cellular reactor. Thus, the interfacial area is determined by the geometric shape of the cellular reactor.

$$S_{LL} = \frac{N \times A_{LL}^{*}}{6}$$

Here, S_{LL} represents the liquid-liquid interface area, N denotes the total number of degrees of freedom for the cellular reactor, and 6 represents the number of faces on a cubic cell (since A_{LL}^{*} is determined by the sum of all faces within the cell). The total area calculated from this is 0.00184 m^2

A comparison of catalytic performance between the use of cellular reactors and the absence of other reactor types. We also consulted the literature and identified a study involving acid-catalyzed addition reaction of dihydrofuran and benzyl alcohol in liquid-liquid reactions, which we compared with our findings.

In terms of catalytic efficiency, a comparative experiment was conducted using 0.1 M H_2SO_4. The reaction volume was 50 mL, with 12.5 mmol of dihydrofuran. At a reaction time of 300 min and a heating temperature of 60°C, the conversion rate reached 97%. In the referenced study, continuous synthesis was carried out using 0.1 M H_2SO_4 with a dihydrofuran concentration of 0.25 M. The reaction was conducted for 500 h at 50°C with a flow rate of 0.6 mL/h, maintaining a conversion rate of 81%. 75 mmol of dihydrofuran was reacted at an 81% conversion rate after 500 h. In contrast, in our work, 75 mmol of dihydrofuran was converted in just 30 h with a yield of 97%.

Supplementary Table 19. Catalytic efficiency between cellular reactor and pickering emulsions

	2H-pyran (mmol)	Time	Yield	Catalyst	Temp.
This work	12.5	3 h	97%	1 M H2SO4	60°C
Reference S1	75	500h	81%	1 M H2SO4	50°C
Reference S2	0.24	18 h	81%	Trialkylphosphonium oxoborate	r.t.
Reference S3	10	1.5	95%	Thiourea, tetrafluoroborate (1:1)	r.t.
Reference S4	5.5	7	98%	Ti^{4+}/4Å	40°C
Reference S5	2	24	88%	Pyridinium	r.t.

Compared to Pickering emulsion[1], the introduction of cellular reactors facilitates rapid reaction occurrence, converting more substrate dihydrofuran at a higher rate per unit time. Unlike Pickering emulsion, which requires rapid agitation for emulsification and subsequent demulsification post-reaction, the introduction of cellular reactors streamlines the operation. Direct use of sulfuric acid as the catalytic component eliminates the synthesis of catalysts[2-5], as well as the post-reaction separation process. Therefore, utilizing cellular reactors for chemical reactions offers unique advantages.

A comparison of extraction performance between cellular reactors and advanced extraction tools. We employed a cellular reactor for liquid-liquid extraction processes. Riboflavin, also known as Vitamin B2, is an essential nutrient for the human body. Initially, riboflavin dissolves in ethyl acetate, imparting a pale yellow color. A 1M NaOH aqueous phase is adsorbed into the cellular reactor. A stirring paddle is inserted into the organic phase and fixed, followed by rotation at a speed of 50 rpm. As stirring progresses, the pale yellow color in the solution gradually lightens, indicating the transfer of riboflavin molecules from the organic phase to the aqueous phase, completing the extraction process.

Using UV-Visible spectroscopy, we measured the riboflavin concentration in the organic phase before and after extraction. The extraction efficiency was calculated as 58.9% (concentration-based) based on the following formula.

$$\eta = \frac{C_0 - C_1}{C_0} \times 100\%$$

In this context, η represents the extraction efficiency, where C_0 denotes the initial concentration and C_0 represents the concentration in the residual phase after extraction.

Compared with state-of-the-art extraction tools, we also reviewed the literature on riboflavin extraction[6]. Riboflavin was successfully extracted using lactic acid ethyl ester and organic salt-based ATPS in the system of lactic acid ethyl ester (1) + sodium citrate (2) + water (3), achieving a maximum efficiency of 87.6%. While the extraction

efficiency of the cellular reactor falls short of previously reported extraction tools, we believe that through modification and optimization, there is potential for expanded applications and improved efficiency in the future.

SI Reference

1 Zhang, M. *et al.* Compartmentalized Droplets for Continuous Flow Liquid–Liquid Interface Catalysis. *Journal of the American Chemical Society*, doi:10.1021/jacs.6b04265 (2016).

2 Das, S., Pekel, D., Neudörfl, J.-M. & Berkessel, A. Organocatalytic Glycosylation by Using Electron‐Deficient Pyridinium Salts. *Angewandte Chemie International Edition*, doi:10.1002/anie.201503156 (2015).

3 Leung, V. M.-Y., Wong, H.-C. F., Pook, C.-M., Tse, Y.-L. S. & Yeung, Y.-Y. Trialkylphosphonium oxoborates as C(sp3)–H oxyanion holes and their application in catalytic chemoselective acetalization. *Chemical Science*, doi:10.1039/d3sc03081d (2023).

4 Magyar, Á., Nagy, B. & Hell, Z. Tetrahydropyranylation of Alcohols in the Presence of a Slightly Basic, Heterogeneous Titanium Catalyst. *Catalysis Letters*, doi:10.1007/s10562-015-1590-8 (2015).

5 Smajlagic, I., Durán, R., Pilkington, M. & Dudding, T. Cyclopropenium Enhanced Thiourea Catalysis. *The Journal of Organic Chemistry*,

doi:10.1021/acs.joc.8b02321 (2018).

6 Velho, P., Oliveira, I., Gómez, E. & Macedo, E. A. pH Study and Partition of Riboflavin in an Ethyl Lactate-Based Aqueous Two-Phase System with Sodium Citrate. *Journal of Chemical & Engineering Data*, doi:10.1021/acs.jced.1c00909 (2022).
