# Supplementary material for: Liquid-liquid reactions performed by cellular reactors
Source: Nat Commun. 2024 Jul 3;15:5579. doi: 10.1038/s41467-024-49953-z (PMC11222485; doi:10.1038/s41467-024-49953-z)
Supplement: Supplementary file 9 — Description of Additional Supplementary Files [file 41467_2024_49953_MOESM9_ESM.pdf]

Description of Additional Supplementary Files

File Name: Supplementary Movie 1

Description: Adsorption of liquids experiment.

File Name: Supplementary Movie 2

Description: Liquid transfer experiment.

File Name: Supplementary Movie 3

Description: Holding liquid experiment.

File Name: Supplementary Movie 4

Description: Interphase mass transfer and chromogenic reaction.

File Name: Supplementary Movie 5

Description: Chromogenic reaction Neutral red discoloration.
